# Development of an automated approach for investigating social learning in mice

**DOI:** 10.3389/fnbeh.2026.1789820

**Published:** 2026-07-15

**Authors:** Benjamin Lang, Christa Thöne-Reineke, Olaf Hellwich, Lars Lewejohann

**Affiliations:** 1 Science of Intelligence, Research Cluster of Excellence, Berlin, Germany; 2 Institute of Animal Welfare, Animal Behavior and Laboratory Animal Science, School of Veterinary Medicine, Freie Universität Berlin, Berlin, Germany; 3 Institute of Computer Engineering and Microelectronics, Faculty IV – Software Engineering and Theoretical Computer Science, Technische Universität Berlin, Berlin, Germany; 4 German Centre for the Protection of Laboratory Animals (Bf3R), German Federal Institute for Risk Assessment (BfR), Berlin, Germany

**Keywords:** animal tracking, home-cage monitoring, IntelliCage, Live Mouse Tracker, mice, social learning

## Abstract

Mice have been shown to learn from each other through social interactions. However, the extent and strategies of social learning in mice remain largely unknown, beyond spatially and temporally limited tests of social memory retention. Here, we present a method that integrates (1) the IntelliCage, a commercially available tool for automated behavioral testing, and (2) the Live Mouse Tracker (LMT), an open-source solution for 24/7 live animal tracking. This approach allows for the investigation of learning behavior in semi-naturalistic group settings while minimizing experimenter interference. In this study, we report on the development of the method, evaluate its accuracy, and identify current limitations. While automated presentation of spatial learning tasks and behavior annotation were effective, identifying individual animals proved unreliable in a highly enriched environment. In response, we provide a rationale for identifying the reliable portion of tracking data, to which we confine the exemplary behavioral analysis. We acknowledge imperfect animal identification as a clear limitation of the method in its current configuration. However, we outline a path to mitigate this and are confident in presenting a promising tool that, after straightforward optimization, may prove useful for various research questions, including the investigation of social learning behavior in mice. The proof-of-principle experiment in this study did not indicate that place learning is facilitated by co-learning over individual learning, and we could not establish a clear association between social interactions and learning performance. While we observed some sporadic differences in interaction rates between co-learning and individually learning animals, we emphasize that these results do not support any conclusions about the mechanisms of social learning in mice. Rather, we present a tool for simultaneously studying learning and tracking behavior with minimal experimenter interference, which, after refinement, may aid future studies of social learning in mice alongside other research questions.

## 1 Introduction

### 1.1 Social learning in mice

Mice (*Mus musculus*) are the most frequently used mammalian species in research (Hickman et al., 2017). Their genetic similarity to humans (Pennacchio, 2003), short generation times, and relatively low housing and maintenance costs (Deacon, 2006) make them a fitting choice for diverse research questions in biomedical research, compound testing, and basic biological and psychological research. In the wild, mice live in large social groups (Berry, 1970) with complex hierarchies (Mackintosh, 1970; Mondragón et al., 1987). Their social lifestyle led to the evolution of mechanisms for the social transmission of information and learning from interactions with conspecifics. These mechanisms are utilized in biomedical research to test for impairments of social behavior associated with novel compounds (Silverman et al., 2013) or transgenic models of autism (Moy and Nadler, 2008). Most prevalent are tests of social amnesia in dyadic or triadic interactions within a designated experimental area distinct from the animals’ home cage, thus being spatially and temporally confined (Stevenson and Caldwell, 2014). Neurologically unimpaired rodents generally display greater interest in unfamiliar conspecifics than in familiar ones. In assays such as the ‘three-chambered social memory test’ (Molosh et al., 2014), decreased social memory retention generally manifests as prolonged exploratory behavior towards familiar animals, which is used to analyze impairments of social behavior.

While the utility of such assays for screening impaired social memory is undisputed, they are limited in the scope of behaviors they can probe. In recent years, advancements in automation and animal tracking have furthered efforts to address the limitations of ‘classical’ dyadic social tests by offering more comprehensive insights into mouse behaviors. Examples of widely used animal tracking solutions include DeepLabCut (Mathis et al., 2018), SuperAnimal (Ye et al., 2022), and LEAP (Pereira et al., 2019) as well as its multi-animal successor SLEAP (Pereira et al., 2022). More recently, Chen et al. (2023) published AlphaTracker, which also offers markerless multi-animal tracking.

The extent to which mice can infer information from their conspecifics remains inconclusive. Mice have shown a preference for food previously consumed by conspecifics in choice tests (Valsecchi and Galef, 1989) and can socially learn fear of aversive stimuli (Jeon et al., 2010). However, documentation of social learning of more complex behaviors is sparse. Few studies have found decreased latency in solving baited mechanical puzzles in mice that observed a demonstrator conspecific compared to naïve mice (Mainardi et al., 1988; Carlier and Jamon, 2006). None of these studies observed true behavioral copying, and all probed social learning in controlled interactions. Studies investigating social learning in more semi-naturalistic group settings are even rarer (Lipina and Roder, 2013). Kiryk et al. (2011) documented an interesting case of social learning in amyloid-β-precursor protein mutant (APP.V717I) mice, which exhibit cognitive impairments similar to Alzheimer’s disease in humans. While these transgenic mice performed significantly worse than their wild-type counterparts in spatial learning tasks, co-housing and co-learning with wild-type mice rescued their spatial learning ability. While the study does not resolve the underlying mechanisms, it suggests that co-learning can mitigate individual learning deficits in mice.

Several neural circuits are recruited in spatial learning paradigms, which approximate group foraging contexts by providing animals with social cues in addition to environmental information (Cheng et al., 2025). The hippocampus has long been established to encode spatial information (Wilson and McNaughton, 1993; Burgess and O’Keefe, 2003; Harris et al., 2003). More recent findings also indicate that hippocampal place-cell ensembles are engaged during observational learning of demonstrator trajectories in rats (Mou et al., 2022). Furthermore, the medial prefrontal cortex, in particular the anterior cingulate cortex, is crucial for mediating social interactions (Kitagawa et al., 2024). Recently, the posteromedial nucleus of the cortical amygdala has been identified as the central node for consolidating socially transmitted food preferences (Liu et al., 2024). Foraging, or baited spatial learning in controlled social contexts, appears to be a multifactorial process recruiting multiple brain regions beyond those listed above, and its underlying neural correlates are yet to be fully understood.

With the increasing availability of multi-animal tracking solutions and automated behavioral testing methods, including those allowing for neural imaging, we anticipate an increase in studies of socially housed groups of mice. This will offer a more comprehensive understanding of murine cognition and behavior (reviewed by Lang et al., 2023).

### 1.2 IntelliCage

**Figure 1:**
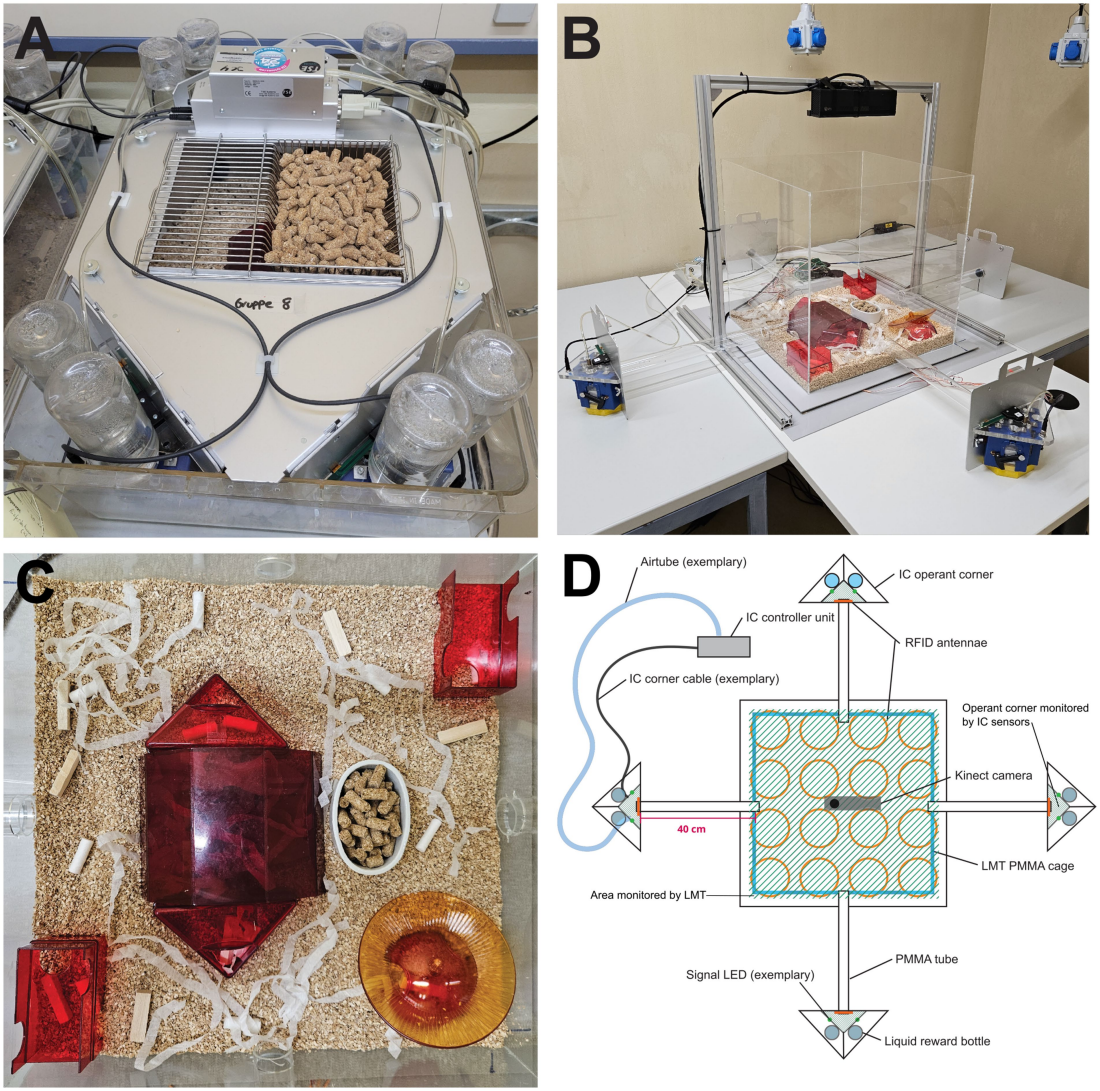
LMT-IC apparatus. **(A)** External view of an IC in its unmodified original configuration. The operant corners were embedded in an aluminum cover with a central food rack inlet, and the IC controller unit was placed atop the cover. Visibility into the cage was largely obstructed in the original configuration. **(B)** Overview of LMT-IC setup with enrichment. Four mice were housed in the central PMMA cage. Liquid reward (4% saccharose in H_2_O) could be obtained from the operant corners placed outside the cage and accessible through 40-cm PMMA tubes. **(C)** Enrichment of the LMT-IC setup, consisting of fine wooden bedding, a main nesting area with three houses, two additional houses in orthogonal corners of the living area, nesting material (paper towels, cotton rolls, and paper strips), wooden gnawing blocks, a running disk, and a millet-filled puzzle ball. Food was offered in a bowl on the bottom rather than in a rack to prevent occlusion from the camera. **(D)** A simplified schematic of the LMT-IC setup from a top-down view. Underneath the floor plate of the PMMA cage, an RFID array of 16 antennae (orange circles) was placed for animal identification via subcutaneously implanted transponders. A depth-sensing infrared camera was placed 63 cm above the cage floor. Each operant corner housed two reward bottles, access to which was regulated via retractable doors. An additional RFID antenna was placed at the entrance of each operant corner. The corners were equipped with sensors for monitoring animal presence, nose-pokes to the doors, and liquid intake, as well as LEDs for visual signal display. Each operant corner was connected to the IC controller unit via cables and tubes for delivering air puffs to the operant corners. The green hatched area indicates the areas covered by monitoring via IR camera, RFID antennae, and the IC operant corner sensors.

The IntelliCage (IC) is a commercially available home-cage-based system for automated behavioral testing of rodents (TSE Systems, Berlin, Germany). It allows for the fully automated presentation of place learning tasks and automated data acquisition, thereby increasing reproducibility by minimizing experimenter interference (Bohlen et al., 2014). The IC consists of a controller unit and four ‘operant corners’ embedded in an aluminum cover (Figure 1A). The entire unit can be placed in a large cage and house social groups of up to 16 animals. Each operant corner contains two bottles that can be filled with liquid rewards (e.g., H_2_O, saccharose solution, almond milk) and accessed through retractable doors. The doors can be programmed to open for individual animals or groups once certain conditions are met. The operant corners are equipped with sensors to identify animals, monitor trials and reward allocation, and present stimuli. Additionally, aversive stimuli can be delivered via pressurized air (air puffs). The system is equipped to perform a wide array of spatial and temporal learning paradigms. However, it offers no way to monitor behaviors and social interactions occurring in the main living area, as all sensors are confined to the operant corners. Furthermore, visual access to the cage interior is severely restricted by the aluminum casing, limiting its applicability to research questions that examine not only learning or memory, but also group interactions.

### 1.3 Live Mouse Tracker

The Live Mouse Tracker (LMT) was developed by de Chaumont et al. (2019) for 24/7 live tracking and automated behavioral annotation of groups of mice, currently up to 4 animals. The method is described in detail in the complementary publication (de Chaumont et al., 2019), as well as on de Chaumont’s website. Briefly, it uses a depth-sensing infrared camera supported by computer vision and machine learning in conjunction with 16 radiofrequency identification (RFID) antennae for 24/7 identification, tracking, and automated behavioral annotation of mice.

Since its introduction in 2019, the LMT has been used to investigate the contributions of autism-associated mutations in the *Shank2* and *Shank3* genes (de Chaumont et al., 2019) as well as the effects of valproic acid exposure on social behavior deficits (Maisterrena et al., 2024). The LMT is primarily distinguished from other multi-animal tracking solutions by its ‘live’ (online) component. It identifies animals, tracks their movements, and annotates behaviors in real time during an ongoing experiment. This allows for real-time review of behavioral data and, if necessary, experimenter intervention (e.g., in case of abnormal behavior indicating impaired animal wellbeing). Furthermore, live tracking enables the integration of third-party devices or software, allowing additional sensors to inform LMT databases, or certain behaviors to automatically trigger responses from external devices (de Chaumont et al., 2019). Behavioral data is stored in highly accessible and easily interpretable SQLite databases. The system foregoes the option to reconstruct animal trajectories from RFID antenna contact mapping because the antennae are selectively activated by the LMT software, and the ability to correct tracking errors is further limited by the low default resolution of the recommended infrared camera.

### 1.4 LMT-IC

Here, we report a method that builds on both the IC and LMT to advance towards fully automated behavioral testing with continuous multi-animal tracking. We aimed to leverage the strengths of both systems while mitigating their limitations by deploying them in concert in a modified setup, which we refer to as the LMT-IC (Figure 1B). By combining the IC’s capacity for automated home-cage-based behavioral tests with the markerless live tracking of the LMT, we created an environment where learning paradigms can be augmented with a vast behavioral dataset. This makes this approach particularly interesting for studies on the interplay of social interactions and learning outcomes. As a proof-of-principle demonstration, we deployed the LMT-IC in a co-learning experiment to examine the putative effect of co-learning scenarios on learning outcomes. We hypothesized that congruent tasks may facilitate the exploitation of unclear, probabilistic reward opportunities, and that animals presented with congruent tasks may display interactions different from those of individually learning animals. The scope of the conducted experiments, in concert with current limitations of the method, however, prevents us from finally confirming or rejecting these hypotheses. Rather, we present a possible future trajectory of investigation with the presented method. With some refinement, the LMT-IC may prove to be a versatile tool for investigating behavior and learning in groups of mice and could be applied to research questions ranging from basic research on cooperation versus competition in a shared-reward landscape to phenotyping disease models or testing novel compounds in semi-naturalistic group settings.

## 2 Methods

### 2.1 Animals

Sixteen female pathogen-free C57BL/6 J mice, aged 42–48 days, were purchased from Charles River Deutschland GmbH, Sulzfeld. Experiments were conducted with two sequential batches (B1 and B2) of 8 mice each. Upon arrival, animals in each batch were randomly divided into two groups (*n* = 4; groups 1 and 2). The four social groups are referred to as B1.1, B1.2, B2.1, and B2.2. Animals were handled using an 11 × 4-cm polymethyl methacrylate (PMMA) tube and trained to enter it voluntarily during the first 2 weeks after arrival.

### 2.2 Animal housing and enrichment

#### 2.2.1 Housing

Animals were kept in a pathogen-free barrier facility at 22 ± 1 °C and 55 ± 1% humidity, under a 12 h light/dark cycle (light phase from 07:00 to 19:00). A wake-up light (Philips HF 3510, 100-240 vac, 50–60 Hz, Philips GmbH Market DACH, Hamburg, Germany) simulated sunrise during the 30 min preceding the light phase. During acclimatization, animals were housed in a Makrolon type III cage (39 × 23 × 15 cm) with *ad libitum* food (LASvendi, LAS QCDiet, Rod 16, autoclavable) and tap water. At 68–74 days of age, they were moved to the LMT-IC experimental setup (Figure 1B) where they resided permanently for the duration of the study. Animals were only briefly removed for weekly cleaning and health checks. During the experimental phase, food (LASvendi, LAS QCDiet, Rod 16, autoclavable) was available *ad libitum,* and tap water/4% saccharose in H_2_O was available via the IC reward bottles.

#### 2.2.2 Enrichment

The LMT-IC apparatus (Figure 1C) featured a 49.2 × 49.2 cm living area, filled with 5 L of fine wooden bedding (JRS Lignocel FS14, spruce/fir, 2.5–4 mm). A main nesting area included three houses (1 × Fat Rat Hut; 16 × 8.6 × 15.2 cm; Bio-Serv, Flemington, New Jersey, USA; 2 × triangular Mouse House; 14.5/11.5 × 5 cm; Tecniplast, Buguggiate, Italy) containing three thin paper towels (cellulose, unbleached, 20 × 20 cm; Lohmann & Rauscher, Neuwied, Germany). In two orthogonal corners, two additional smaller houses (Safe Harbor Mouse Retreat; 6.4 × 11.4 × 7 cm; Bio-Serv, Flemington, New Jersey, USA) were provided, along with a mouse house/running disk combination (house: 10.5 × 5.5 cm, disk: 15 cm; ZOONLAB GmbH, Castrop-Rauxel, Germany). Each cage was supplied with 8 wooden gnawing blocks and additional nesting material: 12 cotton rolls (Gr.3, UNIGLOVES, Troisdorf, Germany) and 30 paper strips (LILLICO, Biotechnology Paper Wool). The PMMA tube used for animal handling remained in the cage. For additional enrichment, a 3D-printed puzzle ball from which the animals could retrieve millet (Alnatura GmbH, Darmstadt, Germany) was offered at two time points (d1 and d21 of the co-learning experiment).

### 2.3 Animal identification

After 1 week of acclimatization to the animal facility (at 49–56 days of age), animals were subcutaneously implanted in the dorsocervical region with radiofrequency identification (RFID) transponders using application cannulas (2.6 × 0.15 × 40-mm ISO-Compliant Transponder; Peddymark Limited, Redhill, Surrey, United Kingdom).

Implantation was performed under isoflurane narcosis (induction: 4% isoflurane CP 1 mL/mL in oxygen at 2 L/min; maintenance: 1% isoflurane CP 1 mL/mL in oxygen at 1 L/min; CP-Pharma Handelsgesellschaft GmbH, Burgdorf, Germany). Approximately 12 h before implantation, animals were orally treated with 0.5 mg/kg Metacam 0.5 mg/mL Suspension (Boehringer Ingelheim, Ingelheim am Rhein, Germany). Additionally, animals were marked with colors (red, green, blue, or yellow; edding 750 paint marker, edding International GmbH, Ahrensburg, Germany) at the tail base for quick visual identification by experimenters and animal caretakers. Markings were maintained weekly.

### 2.4 Live Mouse Tracker

Two Live Mouse Tracker (LMT) apparatuses were constructed as described in detail by de Chaumont et al. (2019). Each apparatus consisted of a custom cage with a living area of 49.2 × 49.2 cm, made from transparent 4-mm PMMA (Firstlaser GmbH, Bardowick, Hamburg, Germany). Beneath the cage, an array of 16 (4 × 4) 100-mm RFID antennae (Priority 1 Design, Port Melbourne, Melbourne, Australia), manually tuned to 134.2 kHz, was installed. A depth-sensing infrared camera (Xbox One Kinect Sensor 2.0, Microsoft Corporation, Redmond, USA) was mounted on a custom-built aluminum rack (Aluminum Profile 30 × 30 L B-Type Groove 8, DOLD Mechatronik GmbH; Haslach, Germany), with one infrared LED masked to avoid overexposure. The system was controlled by the Live Mouse Tracker application (Version 1040). The original design by de Chaumont et al. was modified as follows: (1) the height of the PMMA side panels was increased by 10 cm (from 35 cm to 45 cm) and (2) a circular hole with a 2 cm radius was laser-cut centrally in all four side panels at a height of 4 cm. These were connected by 40 × 4-cm PMMA tubes to the IC operant corners.

### 2.5 IntelliCage

ICs were purchased from TSE Systems (TSE Systems, Berlin, Germany). Two V1.4 models were used in this study. Experiments were controlled by the accompanying IC software V3.3.5.0.

In addition to liquid reward bottles, the IC operant corners are equipped with sensors to detect the presence of an animal, an RFID antenna for identification via subcutaneously implanted transponders, a light-barrier sensor for detecting ‘nose pokes’ (NPs), a ‘lickometer’ for monitoring liquid intake, and three colored LEDs for light signals. The IC Software allows flexible programming to grant or deny reward access and deliver aversive air puffs based on animal identities, operant corner visits, performed NPs, or temporal patterns. It also monitors the animals’ liquid intake from the reward bottles.

### 2.6 Experimental setup

A method was devised to combine the fully automated spatial memory testing capacity of the IC with the 24/7 live tracking of the Live Mouse Tracker (herein, LMT-IC). To achieve this, the simple approach of making the IC operant corners accessible from the 49.2 × 49.2 cm living area of the LMT was chosen. Both systems (IC and LMT) rely on RFID for animal identification. The LMT uses an array of 16 100-mm antennae located below the bottom of the living area, which are sequentially activated to validate or correct individual animal identities. The IC includes one RFID antenna at the entrance of each operant corner, which is constantly active during operation. Due to these designs, the RFID antennae of the LMT and the IC are oriented perpendicularly to each other. The same is true for the electromagnetic fields projected by the antennae, which may result in field interference (Bekkali et al., 2015), preventing the correct reading of transponders within overlapping fields. Preliminary testing revealed the area of interference to be restricted to a distance of <40 cm. Hence, rather than placing the IC operant corners within the living area of the LMT apparatus in a configuration similar to the IC setup (Figure 1A), we opted to place them outside the cage, 40 cm from the outer edge of the living area. The operant corners were connected via PMMA tubes (42 × 4 cm, custom-built from GEHR PMMA XT^®^ ACRYL, Mannheim, Germany), preventing interference from the constantly active IC RFID antennae with the LMT RFID array and allowing for reliable reading of animal transponders. Trials and rewards were recorded by the IC sensors within the operant corners, and animal movements and behaviors were monitored by the RFID grid and the IR camera field of view, which spanned the entire living area of the cage as well as the entrances to the PMMA tubes (Figure 1D). The inside of the PMMA tubes themselves was not covered by the camera field of view or sensors. The internal tube diameter of 3.2 cm, however, physically limited social interactions within them.

#### 2.6.1 3D printing

**Figure 2:**
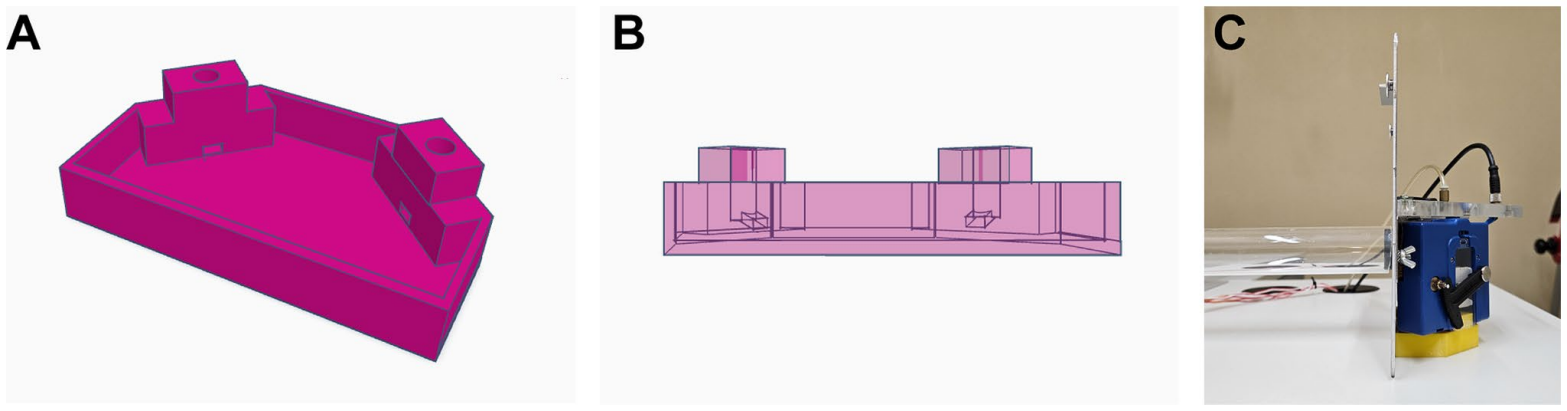
Custom socket for placement of IC operant corners outside of cage area. Custom sockets were designed and 3D-printed to allow connecting the IC operant corners externally to the cage area without the default IC aluminum frame. An internal canal leading into a reservoir allowed for the removal of any excess liquid. **(A)** 3D model of a socket. **(B)** Translucent 3D model of socket with internal canal visible. **(C)** IC operant corner placed in 3D-printed socket (yellow), connected to PMMA tube leading into the living area.

Custom sockets for external placement of the IC operant corners were designed (Figure 2) in Tinkercad (Autodesk, n.d.) and 3D-printed (Ultimaker^3^ Extended; Freeform4U GmbH, Munich, Germany; filament: Polylactic acid (PLA); Filamentworld, Neu-Ulm, Germany). The sockets were designed to ensure stability of the IC operant corners by protruding into the door path of each operant corner. These pins contained an internal canal to allow any excess liquid spilled during liquid consumption by the mice to flow down and be collected in a reservoir at the bottom, preventing liquid accumulation in the apparatus for hygienic reasons.

#### 2.6.2 Custom modifications to the IC

The IC operant corners were removed from the aluminum cover and placed in 3D-printed sockets. The controller unit was unscrewed from the aluminum cover for independent use. All modifications made to the IC were reversible, allowing it to be used in its original configuration in other experiments. To accommodate the larger dimensions of the LMT-based setup, longer cables connecting the IC main controller unit with the operant corners were soldered, and custom tubes (for delivering air puffs to the IC operant corners) longer than the original versions were deployed.

### 2.7 Experimental procedure

The co-learning paradigm was carried out for 41 consecutive days and nights, during which 16 C57BL/6 J mice continuously lived in the LMT-IC apparatus in groups of *n* = 4. Two groups at a time underwent testing simultaneously. Each IC operant corner held two liquid reward bottles, each filled with 250 mL of 4% saccharose in H_2_O. The reward bottles were accessible to the animals during two nightly drinking phases, from 21:00 to 23:00 and 03:00 to 05:00, respectively.

#### 2.7.1 Habituation

Prior to the co-learning paradigm, animals were gradually habituated to the experimental apparatus, the drinking phases, frequenting the operant corners, and performing nose pokes (NPs) to receive a reward. For 24 h, all doors in all operant corners remained continuously open to habituate the animals to visiting them for liquid reward. For another 24 h, all doors in all operant corners would open for all animals upon visiting to habituate them to the door-opening process. Then, animals were gradually habituated to perform an increasing number of 1 to 5 NPs to trigger the door-opening mechanism over the course of a week. In parallel, over the course of 2 weeks, the door opening period was gradually reduced from 20 s to 6 s. Starting in the second week of habituation, mice were also habituated to the nightly drinking phases by first restricting access to the operant corners to the dark phase (19:00–07:00), and subsequently to 21:00–23:00 and 03:00–05:00, respectively. Subsequent to the habituation phase, individual performance in the probabilistic place-learning task was recorded for 2.5 weeks as a baseline measurement before subjecting the animals to the co-learning paradigm.

#### 2.7.2 Rewards and aversive stimuli

During the acclimatization phase, all IC operant corners were baited with plain H_2_O. During baseline measurements and the co-learning paradigm, corners were baited with 4% saccharose (Millipore Ltd., Oakville, Ontario, Canada) in H_2_O. Saccharose was offered to (1) increase animal motivation to participate in the trial, (2) potentially provide additional social cues via saccharose olfaction (Zukerman et al., 2009), and (3) putatively increase affective signaling upon receiving saccharose reward. The 4% saccharose solution was exchanged thrice per week and freshly prepared by stirring until completely dissolved. Two bottles with 250 mL of saccharose solution were deployed per operant corner. Liquid intake was monitored daily. If an animal remained in the same operant corner for >40 s, an air puff (1 s at 0.5 bar) was delivered to that corner to discourage nesting behavior. During the habituation phase, animals were accustomed to the 40-s window by gradually decreasing the time until the air puff was delivered from 180 s to 40 s.

#### 2.7.3 Co-learning experiment

**Figure 3:**
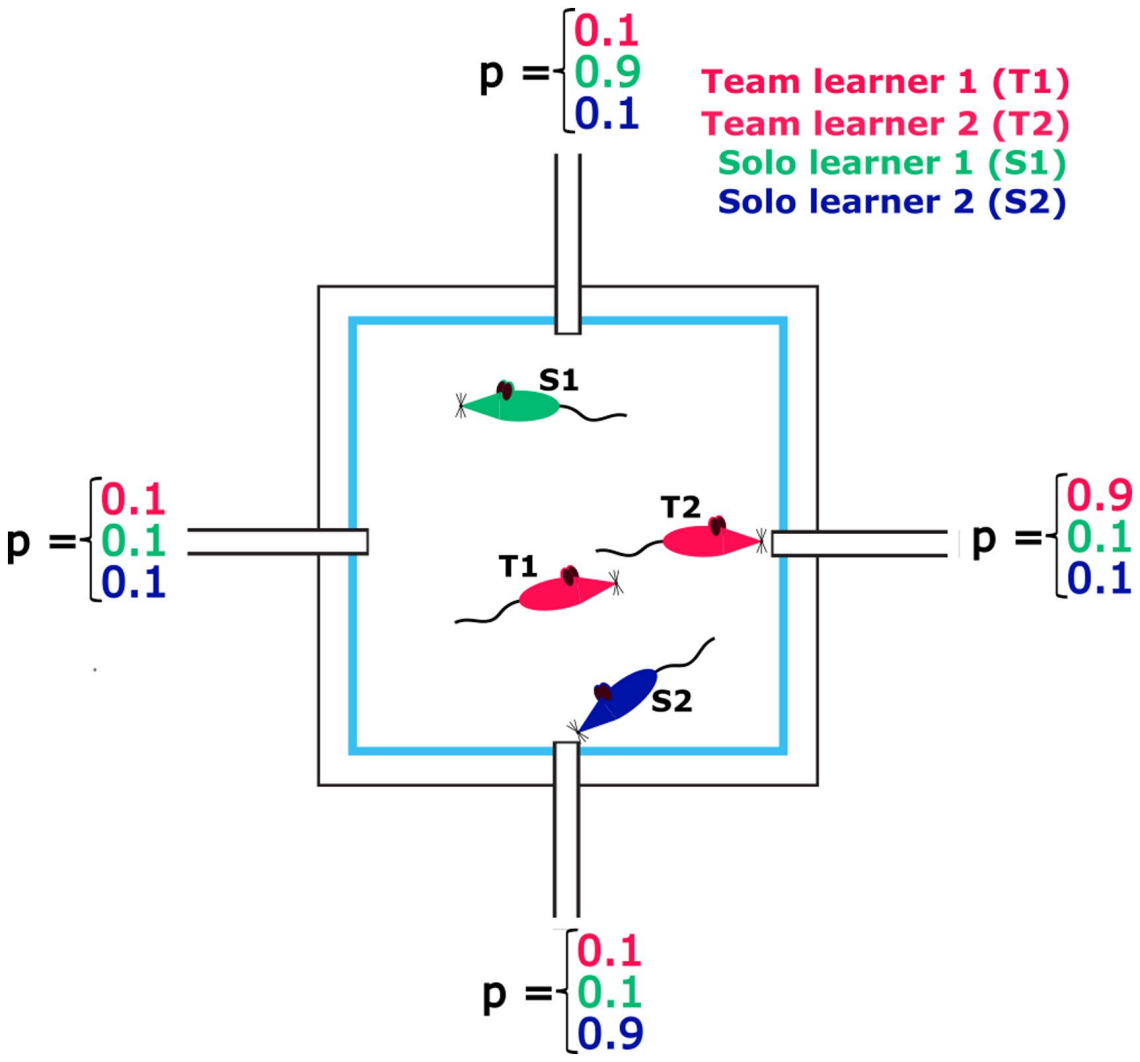
Schematic of reward probability distribution during the co-learning experiment. Per social group, two mice were randomly assigned to a ‘team learning’ configuration (red: animals T1 and T2) and two animals were assigned to a ‘solo learning’ configuration (green: animal S1 and blue: animal S2). At any given time, each animal could obtain a reward at one high-probability operant corner (*p* = 0.9) and at all remaining operant corners (*p* = 0.1). For team animals, the distribution of reward probabilities was congruent, whereas solo-learning animals had individual reward probability distributions. Every 3 days, the distribution of reward probabilities shifted, with both team-learning and solo-learning animals maintaining their respective distributions.

At the start of the co-learning paradigm, animals were randomly assigned to either ‘team learning’ or ‘solo learning’. ‘Team learning’ refers to a co-learning paradigm where two animals per group had the highest probability (*p* = 0.9) of receiving a reward at the same location. The remaining three operant corners each yielded reward with *p* = 0.1. The remaining two animals per group had the highest chance for reward at an individual corner each, not coinciding with the highest reward probability option of any other animal, acting as in-cage controls. To clarify, ‘team learning’ (also referred to as co-learning) describes animals sharing a reward probability distribution across operant corners and does not describe actual enforced cooperation in the learning paradigm. Every third night, the distribution of reward probability options shifted across the operant corners in a semi-randomized manner: a new corner was assigned randomly, excluding the previously assigned corner and any high-reward opportunity operant corner assigned to another solo-learning animal (Figure 3). Low reward probability status was indicated to the animals upon entering a corner by a green LED signal for 1 s. Doors to the reward bottles opened after performing 6 NPs to the light barrier sensor, to which the animals were gradually habituated prior to the start of the experiment. Doors closed after 6 s and would open again only after an animal left the operant corner and began a new trial. Throughout the entire experimental period, animal movement and behavior were tracked via the depth-sensing infrared camera and RFID antennae of the LMT apparatus.

### 2.8 Data processing

#### 2.8.1 IC data

Learning data collected through the IC was analyzed in the open-source statistical software RStudio (Version 2024.04.2 Build 764). For graphical visualization, the ggplot2 (Wilkinson, 2011) package was employed. Descriptive statistics, such as the average number of visits to the operant corners, their average duration, and the latency until the first visits of a night, were computed per animal. Further, an exploratory analysis of sequential trials at the same corner was conducted. For each trial, we examined the probability of a subsequent trial by another animal of the same social group occurring at the same operant corner within 10 s, 30 s, or 1 min. Sequential trials were summarized per dyad across all social groups (Supplementary Figure 1). For statistical modeling, the mixed-effects model package lmerTest (Kuznetsova et al., 2017) was used. Model assumptions were checked visually by Q-Q-plotting and assessing the variance homogeneity of residuals versus fitted values. The model assumed the nightly average preference for the high-reward probability option as the response variable, with the fixed factors (1) social learning configuration (2 levels), (2) night number relative to the reward probability distribution changing (3 levels), absolute night number (32 levels), social group (4 levels), and the individual animals (*n* = 16) as random effect. The overall effects of each fixed factor were evaluated via Type-II *F*-tests with Satterthwaite degrees of freedom (Kuznetsova et al., 2017). For factors with significant effects, estimated marginal means were computed and pairwise comparisons performed with false discovery rate adjustment (Lenth, 2025). Differences are presented with 95% confidence intervals. A bug was discovered in the programming of the semi-randomized assignment of the high probability reward opportunity to animals, which resulted in the faulty assignment of a congruent operant corner as the high reward probability opportunity to the solo-learner mice in one social group (2 animals). The respective nights (*n* = 9) were excluded from analysis and plotting for all animals in the study.

#### 2.8.2 Live Mouse Tracker data

For analysis of live mouse tracking data, scripts provided by de Chaumont et al. (2019) were used, adapted, and modified as needed. Statistical analyses on LMT data were conducted in Python (Version 3.12) and RStudio (Version 2024.04.0 Build 735). The LMT stored tracking data as 10-min video recordings and in SQLite databases. Additionally, the camera provided a three-dimensional depth scan of the cage. For the present study, the tracking and behavioral data in the SQLite databases were utilized. The SQLite database structure is described in detail by de Chaumont et al. (2019). Briefly, for each recorded frame, animal IDs, coordinates, and posture are annotated, along with events (detections, RFID matches/mismatches, social behaviors). Annotated events were reconstructed, and a reliability analysis of the tracking was conducted, which included calculating the rate of animals being unidentified across the total recorded frames and the rates of RFID matches and mismatches upon antenna activation.

#### 2.8.3 Filtering of LMT data

A method was devised to separate detection events that could be directly validated via RFID from possibly erroneous detections. Operations on LMT SQLite databases were based on the scripts provided by de Chaumont (2019a). Initially, events in the SQLite databases that could not be built during live tracking were reconstructed according to de Chaumont (2019a,b). All events for which de Chaumont et al. provided reconstructive scripts were rebuilt, with the exception of (1) Huddling, (2) WaterPoint, (3) WallJump, and (4) SAP (stretched-attend-posture). The computational definitions were adapted unaltered from de Chaumont et al. (2019) and, for the interactions analyzed, are listed in Table 1.

**Table 1:** Behavioral events explanation.

Dyadic event type	Behavioral explanation	Technical definition
Approach	Asymmetric movement of one animal towards at least one other animal.	Distance A ⟺ B decreasing with speed A ≥ B.
Approach contact	Asymmetric approach resulting in contact.	Distance A ⟺ B decreasing with speed A ≥ B, resulting in >3 frames of immediate contact.
Approach rear	An animal (A) is approaching another animal (B) within 2 body lengths distance with B rearing in contact at the conclusion of the approach.	‘Social approach’(A => B) with B ‘rearing in contact’ within ±15 frames of the end of approach.
Break contact	Animals that were previously in immediate contact with each other increase the distance between their centers of mass.	Distance of centers of mass A ⟺ B increases > 45.6 pixels.
Contact	At least two animals (A, B) are in immediate contact with each other.	Distance of centers of mass A ⟺ B ≤ 45.6 pixels.
Get away	At least one animal is moving, with one animal (A) moving faster than the other (B) and consistently increasing distance from each other.	Speed(A) > speed(B); *Δ* t + 1 > Δ *t* − 1.
Group of 2	Two animals in contact with neither of them being in contact with any other animal.	At time *t* ‘Contact’ (A, B) with the exclusion of ‘Contact’(A, C), ‘Contact’(A, D), ‘Contact’(B, C), or ‘Contact’(B, D).
Long chase	Dyadic chase sequences lasting ≥5 s.	Existing ‘Train2’(A, B) events are loaded alongside ‘Contact’(A, B), ‘Break contact’(A⟺B), ‘Escape contact’(A⟺B) and ‘FollowZone’(A⟺B) events. Contact events are extended by ±10 frames whereas all other events are extended by ±5 frames. All ‘Train2’(A, B) events ≥3 frames separated by ≤150 frames are merged and the resulting sequence is extended bidirectionally by ≤300 frames to connect any ‘Contact’(A, B), ‘Break contact’(A⟺B), ‘Escape contact’(A⟺B) and ‘FollowZone’(A⟺B) events within that range. Resulting sequences ≥150 frames are saved as ‘longChase’(A, B) event.
Move in contact	Animal is moving while being in direct contact with at least one other animal.	All detection events during which ‘Contact’, but no ‘Stop’ events take place.
Move isolated	Animal is moving while not being in contact with any other animal.	All detection events during which no ‘Stop’ or ‘Contact’ Events take place.
Nest of 3	Three animals do not move and are in immediate contact with each other, while the fourth animal (A) is not.	Detected ‘stopped’ animals and anonymous detections are checked for contact events and distances between centers of mass ≤45.6 pixels at time *t*. ‘Nest of 3’ occurs when exactly one animal (A) is not in contact with any of the other animals (B, C, D), all of which are in contact with each other and ‘stopped’.
Oral-genital contact	Animal A’s head is in close proximity to animal B’s rear and vice versa.	For each time *t* during which animals A and B are detected, all frames where A’s head is within 15 pixels distance of B’s rear and vice versa, oral-genital contact takes place.
Oral-oral contact	Animal A’s head is in close contact with animal B’s head.	The heads of two detected animals at time *t* are within 15 pixels of each other.
Oral-genital to oral-oral sequence	Oral-genital contact between two animals (A => B) followed by oral-oral contact by the same animals (A, B) within 2 s.	For each ‘Oral-genital contact’ event between a dyad (A, B) with ≥10 frames duration, the subsequent 60 frames are scanned for ‘Oral-oral contact’ events of the same dyad with ≥10 frames duration and, if found, fused into ‘Oral-genital to oral–oral sequence’ from the first frame of the ‘Oral-genital contact’ to the last frame of the ‘Oral-oral contact’ event.
Oral-oral to oral-genital sequence	Oral-oral contact between two animals (A, B) followed by oral-genital contact by the same animals (A => B) within 2 s.	For each ‘Oral-oral contact’ event between a dyad (A, B) with ≥10 frames duration, the subsequent 60 frames are scanned for ‘Oral-genital contact’ events of the same dyad (A, B) with ≥10 frames duration and, if found, fused into ‘Oral-oral to oral–genital sequence’ from the first frame of the ‘Oral-oral contact’ to the last frame of the ‘Oral-genital contact’ event.
Rear in contact	Animal is rearing while in contact with another animal.	Body slope ≥ 40° at time *t* with concurrent contact event.
Rear isolated	Animal is rearing while not in contact with any other animal.	Body slope ≥ 40° at time *t* without concurrent contact event.
Side-by-side contact	Two animals are in close parallel contact.	Two animals including their heads and tails are both detected at time *t* with body vector scalar product ≥ 0 and both heads and tails within a 15-pixel distance of each other, respectively.
Side-by-side contact in opposite orientation	Two animals are in close antiparallel contact.	Two animals (A, B) including their heads and tails are both detected at time *t* with body vector scalar product < 0 and A’s head within 15 pixels of B’s rear and vice versa.
Social approach	One animal approaching another while being within 2 average body-length distances of each other.	Approach events A => B occurring within 0 ≤ dist(A, B) ≤ 2 × meanBodyLength(B).
Social escape	One animal moving away from another while being within 2 average body-length distances of each other.	‘Get away’ events occurring within 0 ≤ dist(A, B) ≤ 2 × meanBodyLength(B).
Stop in contact	Animal A is stopping (not moving) while being in direct contact with at least one other animal.	All overlapping frames between ‘Stop’(A) and ‘Contact’(A, B) at time *t*.
Stop isolated	Animal A is stopping (not moving) while not being in contact with any other animal.	All frames where ‘Stop’(A) takes place without concurrent ‘Contact’(A, B) event or no other animal except A detected.

The table lists the behavioral events investigated within the social learning experiment. Each event type (1st column) is given a behavioral explanation (2nd column) alongside a brief technical definition (3rd column).

**Figure 4:**
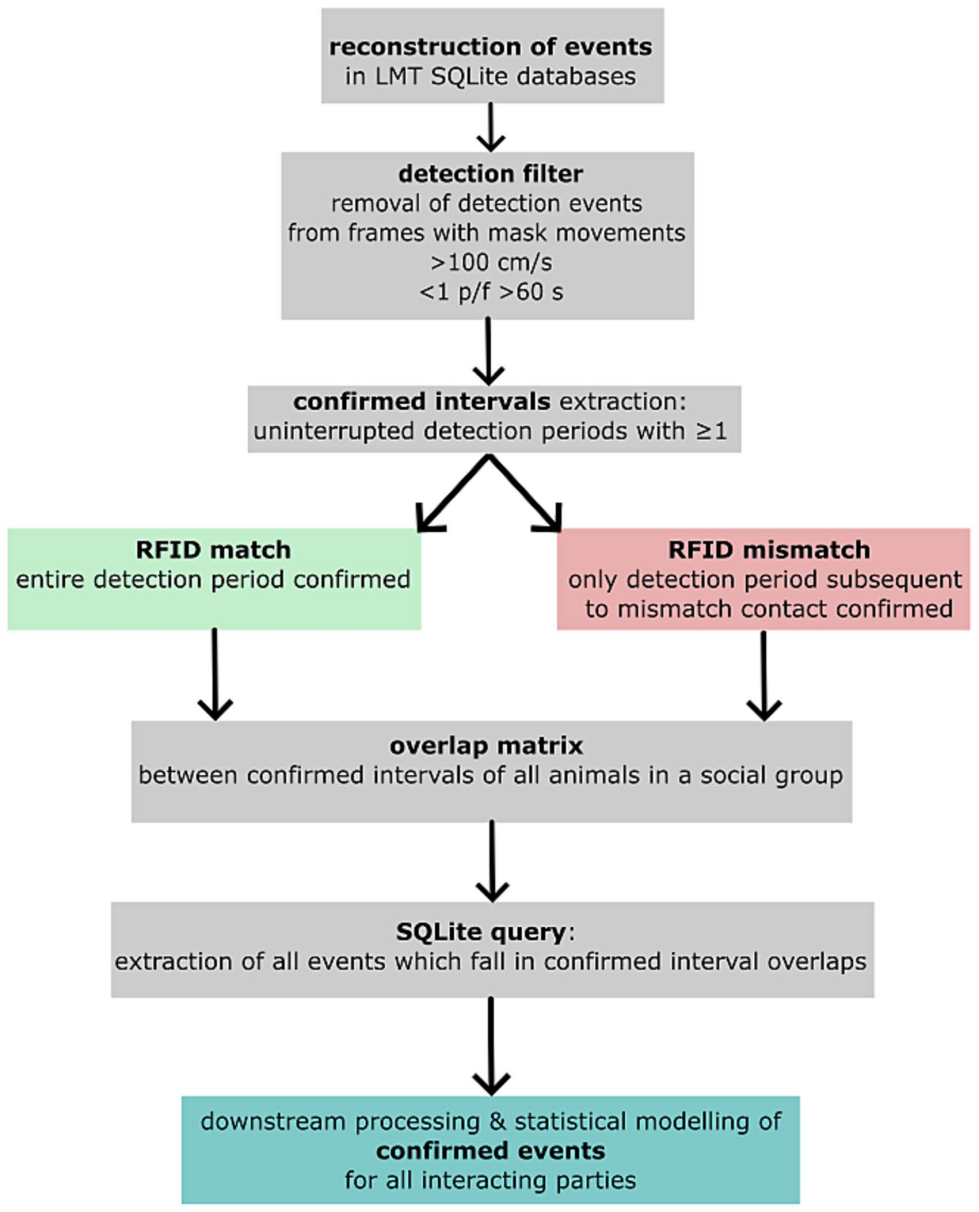
Schematic of LMT data filtering pipeline. Events were reconstructed in the raw SQLite databases. Filtering was implemented in 2 stages: first, mask movements were limited to ≤100 cm/s and >1 pixel per consecutive frame (p/f) < 60 s. Frames with mask movements of any animal exceeding these thresholds were deprived of the ‘detection’ label. In the second step, of the remaining detection periods, only those were retained that represented continuous, uninterrupted, unoccluded detections containing at least one RFID event. For RFID match events (i.e., the LMT-assigned identity was confirmed by RFID antenna activation and reading of the animal’s RFID transponder), the entire uninterrupted detection period was confirmed. For RFID mismatch events (i.e., the LMT-assigned identity was revealed to be erroneous by RFID antenna activation and reading of the animal’s RFID transponder, resulting in the correction of the previously assigned animal identity), only the portion of the period after the ID correction was confirmed. Of the confirmed detection periods, overlaps across all animal combinations were identified, and interactions of the respective combinations were extracted via SQLite query to determine the subset of interactions and behaviors for which all involved parties could be unequivocally assigned an ID.

The SQLite databases with reconstructed events were used for overall detection analysis at the group level. For analysis of individual animal behaviors, the following set of restrictions was applied to isolate portions of the data that allowed for unambiguous assignment of animal IDs. The filtering process is detailed in Figure 4.

‘Confirmed detection intervals’ were identified for each animal, and subsequently, the overlapping frames of those intervals were determined for all possible combinations of animals per social group. Then, the LMT SQLite databases were queried, and only events that fell within these overlaps of confirmed presence among all interacting parties were considered (herein, ‘events in confirmed intervals’). To account for potential imbalances in confirmed detection periods, the total confirmed co-presence was calculated per animal dyad, and interactions were presented as interaction rates per confirmed co-presence in frames.

#### 2.8.4 Validation of the LMT-IC system

The retention of data in frames subsequent to each filtering step was calculated in absolute frames with animal detections, as well as the percentage of the detection count in the raw data (Table 2). Furthermore, the frequencies of intervals of confirmed co-presence of varying duration in frames were visualized in histograms on a log_10_(*n* + 1) scale.

**Table 2:** Frame retention during the LMT data filtering process.

Processing stage	Metric / Animal	B1.1				B1.2				B2.1				B2.2			
		T1	T2	S1	S2	T1	T2	S1	S2	T1	T2	S1	S2	T1	T2	S1	S2
Raw data	Recorded (f)	143,090,679	143,090,679	143,090,679	143,090,679	144,737,231	144,737,231	144,737,231	144,737,231	145,460,125	145,460,125	145,460,125	145,460,125	137,955,292	137,955,292	137,955,292	137,955,292
	Detected (f)	35,022,060	34,626,430	35,991,039	36,966,176	56,571,761	56,362,464	56,405,011	56,522,420	38,306,763	36,965,764	37,843,750	39,992,085	55,766,746	57,793,644	59,049,073	58,090,265
	Detected (%)	24.48	24.2	25.15	25.83	39.09	38.94	38.97	39.05	26.33	25.41	26.02	27.49	40.42	41.89	42.80	42.11
	Match (*n*)	19,808	28,610	69,087	73,630	95,978	80,869	77,795	79,166	62,782	23,887	27,324	111,283	91,565	160,206	188,930	157,465
	Mismatch (*n*)	1,097	1,087	957	737	956	1,007	1,022	1,055	2,601	2019	2,300	2,399	1806	1839	1,444	1,577
Speed/stat. filtered	Recorded (f)	143,090,679	143,090,679	143,090,679	143,090,679	124,187,348	124,187,348	124,187,348	124,187,348	145,460,125	145,460,125	145,460,125	145,460,125	137,955,292	137,955,292	137,955,292	137,955,292
	Detected (f)	25,630,071	25,211,592	26,751,826	27,877,320	39,361,270	39,822,623	38,890,652	38,977,898	29,930,397	28,905,298	29,405,176	31,929,573	40,499,138	41,278,619	41,912,087	40,946,020
	Total retained (%)	17.91	17.62	18.70	19.48	31.70	32.07	31.32	31.39	20.58	19.87	20.22	21.95	29.36	29.92	30.38	29.68
	Detections retained (%)	73.18	72.81	74.33	75.41	69.58	70.65	68.95	68.96	78.13	78.19	77.70	79.84	72.62	71.42	70.98	70.49
	Match (*n*)	19,808	28,610	69,087	73,630	93,043	76,979	71,226	71,935	62,782	23,887	27,324	111,283	91,565	160,206	188,930	157,465
	Mismatch (*n*)	1,097	1,087	957	737	868	934	899	958	2,601	2019	2,300	2,399	1806	1839	1,444	1,577
RFID match/mismatch filtered	Recorded (f)	143,110,162	143,110,162	143,110,162	125,732,659	124,187,348	124,187,348	124,187,348	124,187,348	145,460,125	145,460,125	145,460,125	145,460,125	137,955,292	117,515,116	137,955,292	137,955,292
	Detected (f)	1,215,144	1,385,441	2,668,802	3,283,060	2,230,688	2,328,724	2,001,955	2,059,599	2,157,733	1,618,830	1,614,050	3,607,080	2,544,253	2,721,915	4,666,747	3,951,943
	Total retained (%)	0.85	0.97	1.86	2.61	1.80	1.88	1.61	1.66	1.48	1.11	1.11	2.48	1.84	2.32	3.38	2.86
	Detections retained (%)	3.47	4	7.72	8.88	3.94	4.13	3.55	3.64	5.63	4.38	4.27	9.02	4.56	4.71	7.9	6.8
	Match (*n*)	883	925	2,908	4,710	4,100	4,030	2,774	3,812	2,464	1,256	1,637	5,937	2,778	7,918	8,166	6,806
	Mismatch (*n*)	725	732	569	429	472	536	533	556	668	679	701	406	1,135	829	795	884
Confirmed co-presence (f)	T1		119,079	196,581	299,823		208,810	194,867	184,070		199,691	218,979	306,000		199,691	218,979	306,000
	T2	119,079		222,622	272,313	208,810		222,029	196,530	199,691		156,413	274,197	199,691		156,413	274,197
	S1	196,581	222,622		458,126	194,867	222,029		185,660	218,979	156,413		229,737	218,979	156,413		229,737
	S2	299,823	272,313	458,126		184,070	196,530	185,660		306,000	274,197	229,737		306,000	274,197	229,737	

The table lists the total number of frames recorded per animal during co-learning experiments and baseline measurements (Recorded (f)), the number of frames during which animals were detected in the raw LMT data (Detected (f)), and the percentage of total recorded frames covered by animal detections (Detected (%)). It also includes the number of RFID match and mismatch events (Match (*n*), Mismatch (*n*)) per social group (B1.1, B1.2, B2.1, B2.2). The same metrics are given for each filtering step (Speed- and stationarity filter, Match and mismatch filter), where ‘Detected (f)’ denotes the number of frames with detection events remaining after the filtering step. The remaining detection events are also given as percentages of the total recording duration in frames (Recorded (f)) and of the raw detection frames (Detections retained (%)). The confirmed co-presence between animals is presented as an overlap matrix of the confirmed co-presence durations in frames.

To assess whether the strict tracking data inclusion criteria led to a spatial bias in data retention, the camera field of view, covering the entire living area, was divided into a grid of 100 × 100 bins. The number of total detections per social group falling into each bin was calculated for both the raw and the filtered datasets. 2D heatmaps were generated depicting the absolute number of detections per bin as well as normalized occupancy maps. For the occupancy maps, the fraction of total detections falling into each bin was calculated for both the raw and filtered datasets to allow for direct visual comparison of the spatial distribution of animal detections before and after applying the strict data inclusion criteria. Per social group, the global keep rate was calculated as the total number of retained detections divided by the total number of detections in the corresponding raw dataset. Spearman rank correlations were calculated across the 100 × 100 bin grid using the normalized occupancy values within the filtered and raw datasets, respectively.

To assess whether the congruent reward probability distributions in team-learning animals resulted in a spatial bias in confirmed co-presence (and by extension, dyadic interactions) towards the PMMA tube entrances leading to the IC operant corners, we localized the intervals of confirmed co-presence to three coarse spatial categories. For each frame of confirmed co-presence between all dyads, the center of mass coordinates were retrieved from the SQLite databases and localized to either ‘tube proximity’ (a radius of 7.5 cm around the tube entrances), ‘center’ (a 34.56 × 34.56-cm central square in the cage area), or ‘periphery’ (the remaining marginal areas not covered by the ‘tube proximity’ radii). Co-present dyads were classified as team-team (T–T), team-solo (T-S), or solo-solo (S-S) based on their assigned learning configuration, and the frames with confirmed co-presence for each dyad category were localized to the three spatial categories. We fitted generalized linear mixed models for each localization response. The number of confirmed co-presence frames localized to tube proximity was modeled against the remaining confirmed co-presence frames, assuming dyad configuration (T–T, T-S, S-S) as a fixed effect and social group (B1.1, B1.2, B2.1, B2.2) as a random intercept, both for instances of at least one co-present party being localized at a tube entrance, as well as both parties of a dyad being co-present at the same tube entrance. Model dispersion was assessed using the DHARMa package (Hartig, 2026). Estimated marginal means were back-transformed to the response scale and are reported as probabilities with 95% confidence intervals with Tukey-adjusted pairwise comparisons between dyad configurations.

#### 2.8.5 Statistical modeling of dyadic interactions

For statistical modeling of dyadic social interactions, carried out in RStudio (Version 2024.04.2 Build 764), the lmerTest (Kuznetsova et al., 2017) package was used. Model assumptions were visually checked by Q-Q-plotting and by assessing the variance homogeneity of residuals versus fitted values. The model used average dyadic interaction density (average dyadic interaction time in frames per confirmed co-presence in frames) as the log-transformed response variable to accommodate the strictly positive, right-skewed interaction density. The model included dyadic event type (20 levels), social learning configuration interaction type (3 levels), their interaction, and social group (4 levels) as fixed factors, with the interacting dyad included as a random intercept (24 levels). As the baseline measurement and social learning experiment differed in both length and learning tasks, we modeled the data from both phases separately, applying the same model assumptions. This approach was further supported by a highly significant effect of the learning configuration × experimental phase interaction (*F* = 10.4; *p* = 3.52 × 10^−05^) when expanding the model across both phases. The overall effects of fixed factors were assessed via Type-III *F*-tests with Kenward–Roger degrees of freedom (Kuznetsova et al., 2017). For factors with a significant effect, estimated marginal means were computed, and Tukey-adjusted pairwise comparisons were performed (Lenth, 2025). Estimated means and differences are presented on the original scale with 95% confidence intervals.

#### 2.8.6 Correlation

For each animal, total nightly participation (according to the conservative inclusion criteria; ref. Figure 4) in LMT events ‘Approach’, ‘Approach contact’, ‘Approach rear’, ‘Break contact’, ‘Contact’, ‘Get away’, ‘Group of 2’, ‘Move in contact’, ‘Move isolated’, ‘Nest of 3’, ‘Oral-genital contact’, ‘Oral-oral contact’, ‘Rear in contact’, ‘Rear isolated’, ‘Side by side contact’, ‘Side by side contact in opposite orientation’, ‘Social approach’, ‘Social escape’, ‘Stop in contact’, ‘Stop isolated’ was summed (irrespective of interaction partners) per dark phase (19:00–07:00), and checked for monotonic association with the preference for the high-reward probability opportunity during the same time periods using Spearman’s rank correlation. Only behaviors with ≥10 nights having both non-zero event durations and visit counts were included. Spearman’s Rho (herein, *ϱ*) was calculated for the aligned pairs via the R 4.2 base package ‘stats’ (R Core Team, 2023) alongside the corresponding *p*-values using non-parametric percentile bootstrap (2000 resamples).

## 3 Results

### 3.1 Validation of the LMT-IC system

#### 3.1.1 Animal detection coverage

**Figure 5:**
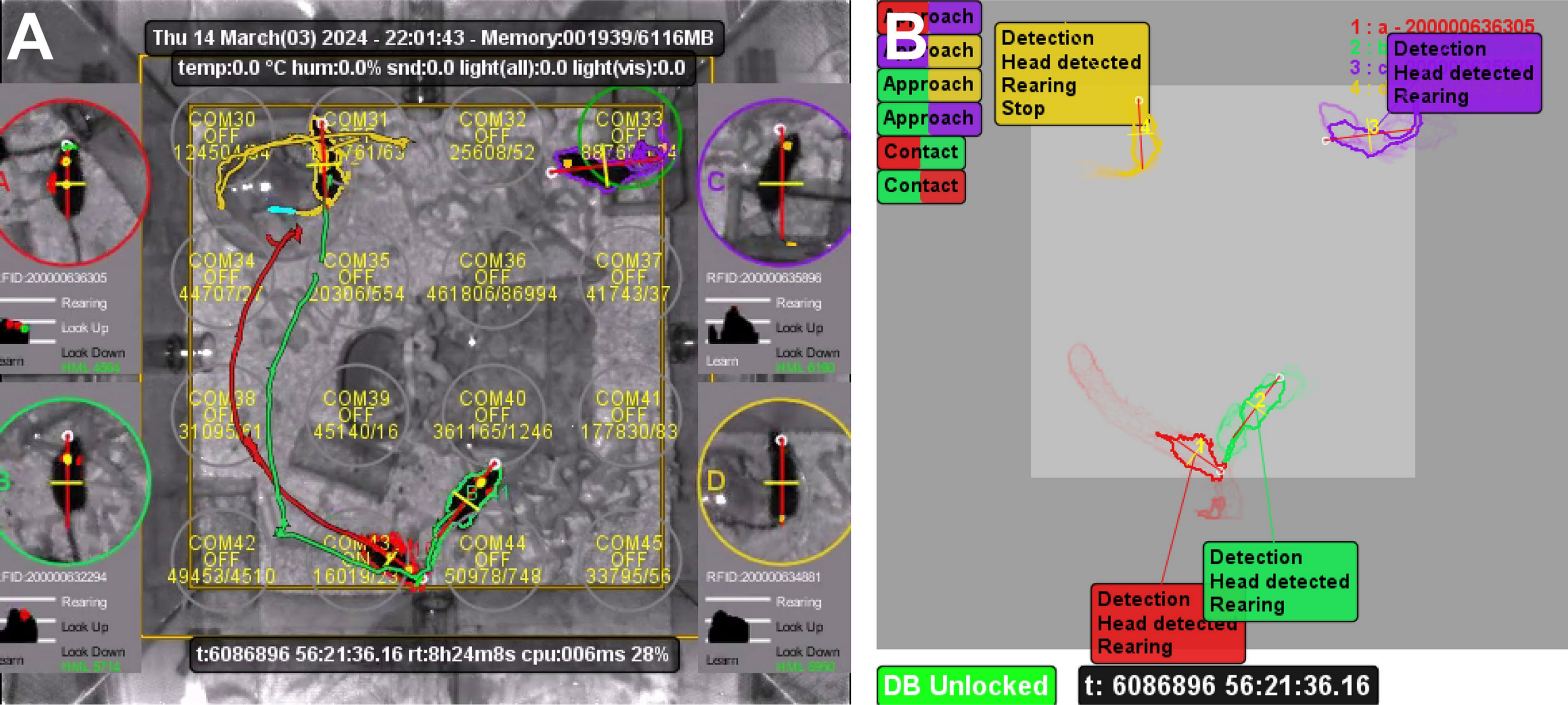
Live mouse tracking. **(A)** Top-down camera view into the cage. Capture taken 1 h after the onset of the dark period, with the four mice present in the field of view. Colored masks outline individual animals and recent trajectories. Animal 1 (red) is in contact with animal 2 (green), while animal 3 (purple) is inside the top right corner house, facing animal 4. Animal 4 (yellow) is running on the running disk. **(B)** Tracking view of the same time point as in **(A)**. Text overlays next to animal masks detail detection and pose estimate. In the top left corner, social interactions (here: approach and contact) occurring in the current frame are detailed, with color-coding representing the animals involved.

We report on the development of a novel approach to tracking mouse behavior and learning performance over extended periods with minimal experimenter interference by building on existing approaches—the LMT and the IC. We first assessed the tracking accuracy achievable by deploying the two systems in concert, before presenting the investigation of social learning in mouse groups as a proof-of-principle application. To that end, mice (*n* = 16) were continuously housed in the LMT-IC system for 41 days while being presented with probabilistic reward options through the IC. During this time period, the mice were continuously tracked by the LMT component of the system, which also automatically annotated individual and social behaviors (Figure 5; for a list of currently available behaviors detectable by the LMT, see de Chaumont et al., 2019).

We first evaluated the tracking coverage and animal identification precision of the LMT. Whereas the automated annotation of behaviors (see Table 1) was highly accurate compared to behavioral assessment by experimenters, detection and identification of the animals proved imperfect in the current setup configuration.

**Figure 6:**
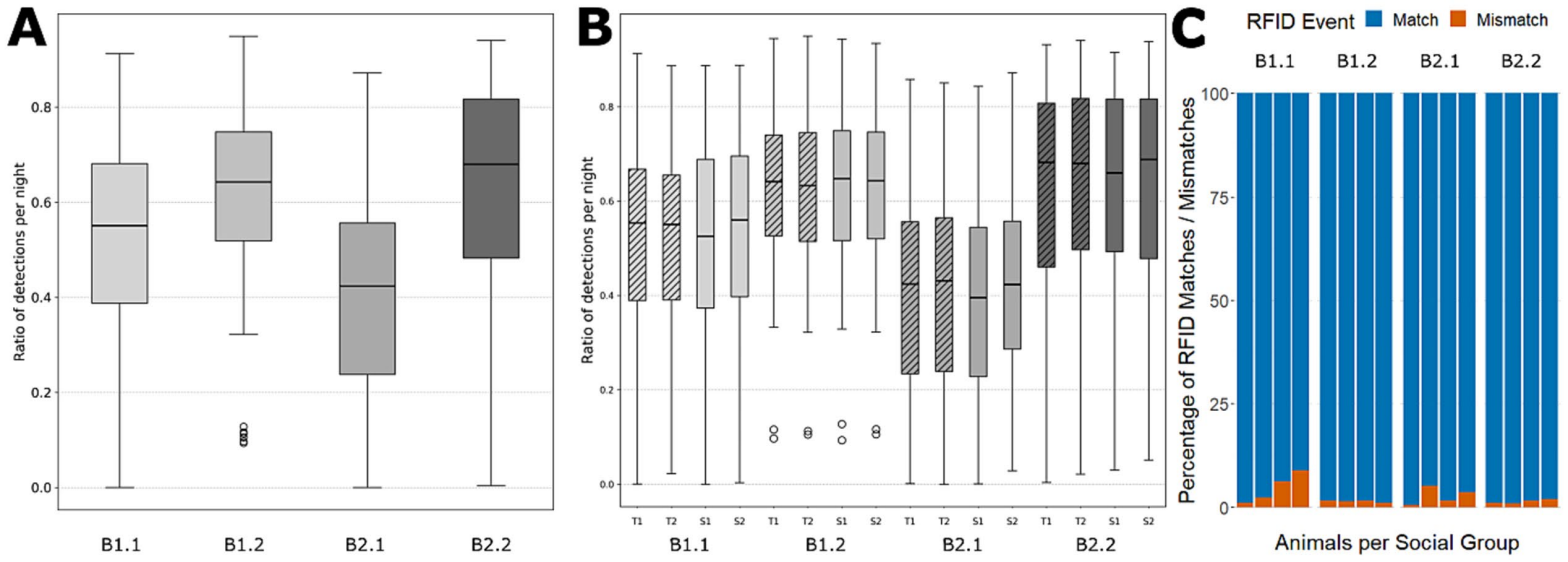
LMT detection accuracy. **(A,B)** Animal detection rates during the dark phase (19:00–07:00). Boxplots show the average detection rates across all nights in the experiment, by social group and animal. Each boxplot **(A)** and group of subplots **(B)** depicts data for 4 animals over ~75 nights. **(A)** By social group (*n* = 4). **(B)** By individual animals (*n* = 16). Hatched plots represent co-learning animals; unhatched plots represent individually learning animals. **(C)** Percentage of RFID verification/correction (match/mismatch) during tracking. One RFID antenna is always active to either identify unidentified animals or validate the ID of presumably identified animals. The blue portion of the graph depicts the percentage of instances where the presumed identity of an animal was validated upon antenna activation, and the orange portion depicts the percentage of instances where the animal ID was corrected upon antenna activation.

Overall, mice were detected in half of all frames during the dark phases (Figure 6A; 0.543 ± 0.232). Notably, the average detection rates of social groups B1.1 and B2.1 were slightly lower (0.516 ± 0.221 and 0.406 ± 0.223, respectively) than those of the two other social groups B1.2 and B2.2 (0.625 ± 0.173 and 0.626 ± 0.224, respectively). One LMT-IC system was used for groups B1.1 and B2.1, whereas another was deployed for groups B1.2 and B2.2, possibly suggesting a performance difference in the hardware. Individual detection rates (Figure 6B) mirrored the detection rates at the social group level, with considerable intergroup variance (Ø ± 0.21) but little intragroup variance (Ø ± 0.006). The rate of dark-phase animal detection here is averaged over the entire study duration, which included periods of animal inactivity. During these periods, particularly after the first trial phase ended, the animals commonly remained huddled in the nesting area, where they could not be segmented by the LMT. In these scenarios, identification could not be rescued by activation of RFID antennae, as the antenna grid resolution is not high enough to discriminate huddled, stationary mice (Supplementary Figure 2). The detection rate was highest during the first trial phases of the night (21:00–23:00), when the animals were most active (Supplementary Figure 3).

We attribute the imperfect animal identification mainly to the very high degree of enrichment used in the study, which led to occlusion of animals from the camera’s field of view. The rate of RFID corrections (instances in which an animal is supposedly identified by the tracking algorithm but then identity-corrected upon RFID antenna activation) was low (overall 2.71%; Figure 6C). This rate was similar to the misidentification rate reported by de Chaumont et al. (2019) (2.69%) and was calculated using the same method provided by de Chaumont et al. This suggests that once animals are visible and can be segmented by the machine learning algorithm, identification is relatively stable.

#### 3.1.2 Misidentification in a highly enriched environment

A current limitation of the LMT-IC system in a highly enriched living area is the temporary misidentification of animals. This occurs when an animal is occluded by enrichment or multiple animals frequent the same operant corner within a short time period. The high degree of enrichment deployed in our study, coupled with the ability of the animals to remove themselves from the camera’s field of view at any time, likely interfered with consistent animal identification. This manifested as the video-tracking component ‘losing’ identified animals between RFID antenna contacts, or assigned IDs being swapped among detection masks. De Chaumont et al. (2019), who used a small custom-built mouse house as the sole enrichment in their study, did not report such difficulties in maintaining continuous animal ID assignment.

To mitigate animal misidentification, strict filters were imposed on the tracking data. For the exemplary analysis of interactions assigned to solo- and team-learning configurations, we opted for a very conservative identification method (Figure 4) by only considering instances where animal IDs could be directly confirmed by reading their respective RFID tags, thereby omitting the majority of the data (Table 2).

**Figure 7:**
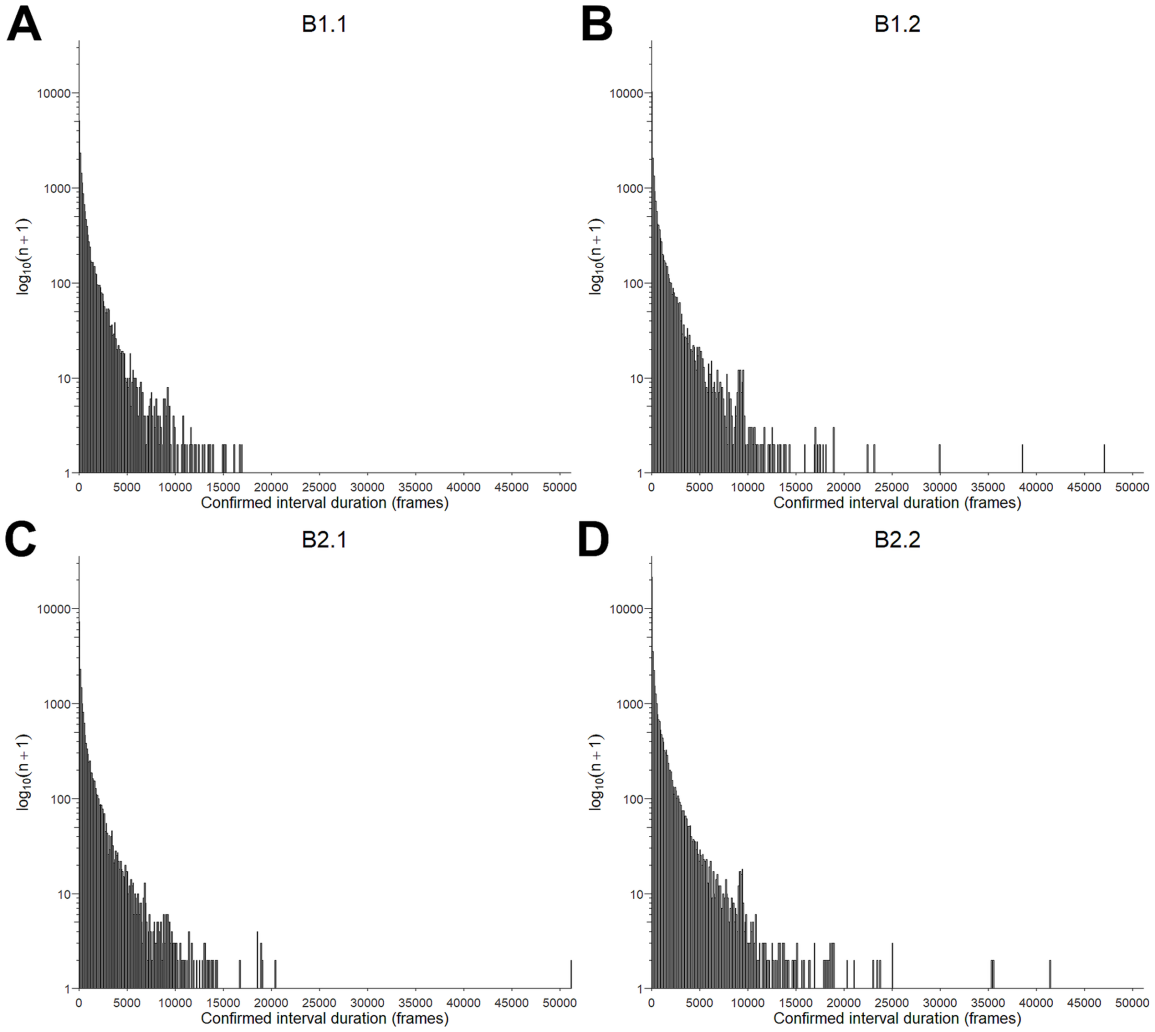
Histograms of confirmed detection intervals per group. Plots depict the frequency of detection intervals in relation to their duration, sorted by group. Frequency is expressed on a logarithmic scale for better readability. The occurrence of all intervals was increased by 1 before log transformation (log10(*n* + 1)) to include intervals that occurred only once in the plot. **(A)** B1.1, **(B)** B1.2, **(C)** B2.1, and **(D)** B2.2.

The ‘confirmed detection intervals’ ranged from 1 to 51,173 frames in length, with the distribution heavily skewed towards short intervals (Figure 7). This was partially accounted for when selecting the linear mixed model, assuming interaction density per confirmed co-presence as the response variable. However, the filtering process inherently favored shorter interactions, with the probability that a ‘confirmed’ detection period remained uninterrupted by identification errors decaying exponentially with interval length. For all subsequent analyses, the total interaction durations for all occurrences of an interaction type per time unit were considered rather than the durations of individual interactions.

**Figure 8:**
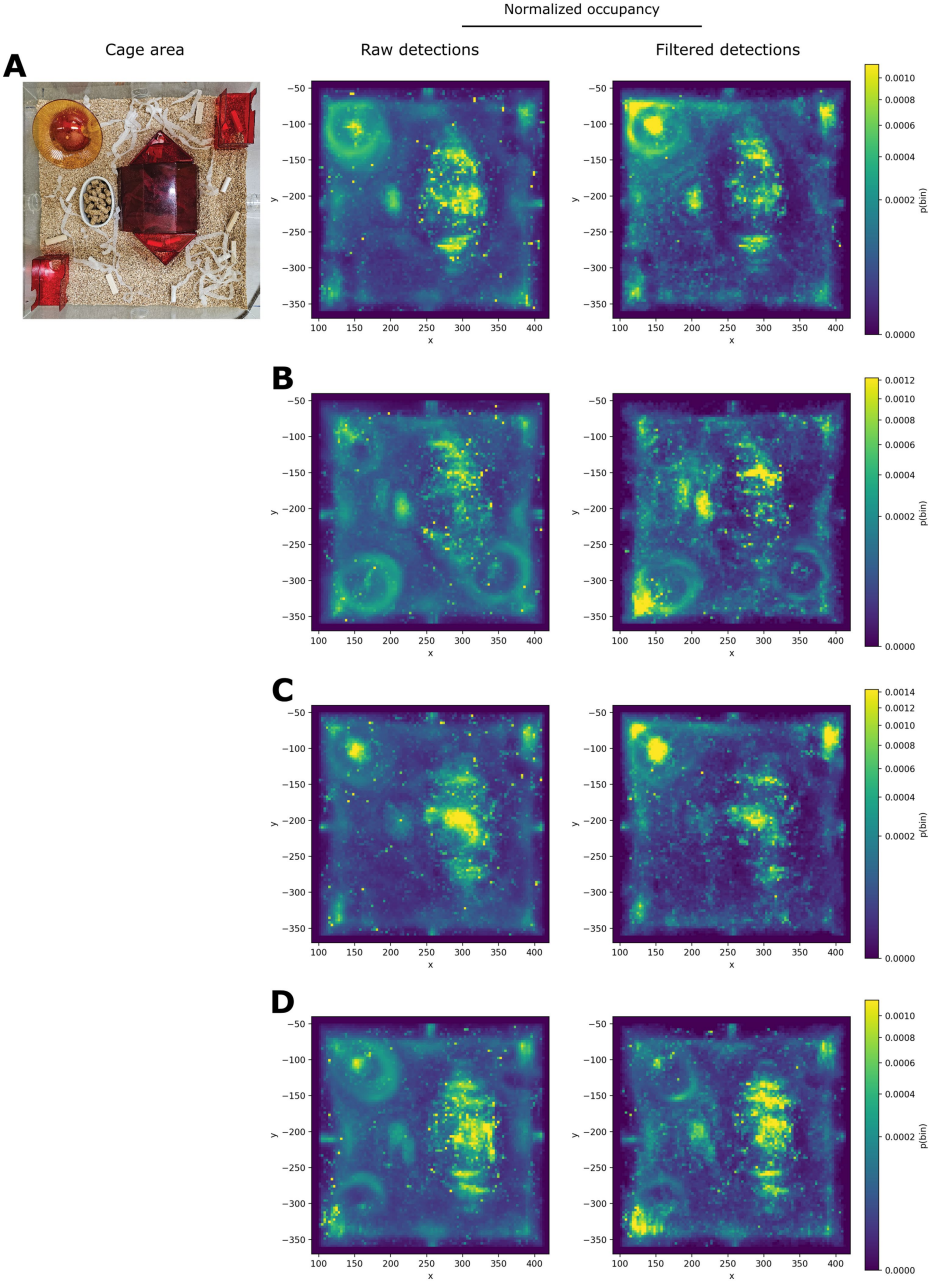
Relative distribution of animal detections in raw and filtered LMT data. All detections per social group were pooled across the entire duration of the co-learning experiment. The LMT field of view was divided into 100 × 100 bins. For each bin, the fraction of total detections falling into that bin was calculated and plotted both before (middle panel) and after subjecting the dataset to the filtering pipeline (right panel). Relative detection densities were plotted on the same color scale for visual comparability. **(A)** B1.1, alongside the cage structure for spatial reference, **(B)** B1.2, **(C)** B2.1, and **(D)** B2.2.

The highly conservative inclusion of detection periods inevitably substantially reduced the LMT dataset and introduced the possibility of spatial filtering effects. To assess whether particular cage areas were differentially impacted by the filtering process, all animal detections in both the raw LMT data and the filtered subset of ‘confirmed intervals’ were binned into a 100 × 100 grid across the cage living area. The absolute detection counts per bin (Supplementary Figure 4) revealed a similar distribution of retained detections in the filtered dataset to that in the raw data, albeit with signal strengths lower by orders of magnitude. To enable direct visual comparison and more accurate investigation of potential spatial filter effects, we also created normalized occupancy maps of the raw and filtered detection datasets (Figure 8) by calculating the fraction of all detections per bin and plotting them using the same color scaling for raw and filtered detection datasets. The global keep rate across social groups was 7.11 ± 2.17% of total detections, which is consistent with frame retention analysis during filtering (Table 2).

Across social groups, the filtered detection subset preserved the broad spatial structure of the detection distribution in the raw dataset. The Spearman rank correlation between the detection densities per bin in the filtered and raw datasets was 0.84 ± 0.02 on average, indicating a high degree of concordance in the relative spatial distribution of detections. Visual inspection of occupancy maps suggested spatial distributions of detections were largely preserved, retaining major detection ‘hotspots’ from the raw to the filtered datasets, such as the central nesting area, food dish, side houses, running disk, and tube entrances leading to the IC operant corners (Figure 8). Areas with below-average detection retention appear to mainly correspond to the cage edges and the sloped walls of the large central house. We conclude that while the filtering pipeline strongly reduced the volume of the LMT dataset, it did not substantially alter the spatial distribution of detections.

Overall, the LMT-IC system showed promise in its ability to track animals over long periods while also subjecting them to behavioral testing. For research questions that rely on individual animal identification, the error rate in ID assignment proved inadequate in settings where animals can remove themselves from the LMT field of view. To illustrate its capacity to serve as a tool for behavioral research, we implemented a rigorous filtering pipeline that prioritized identity certainty over data conservation. This strict data selection regime left us with only a tiny subset of the original data, which, however, we could demonstrate was relatively evenly distributed and did not introduce any pronounced inherent spatial bias by the filtering method. As proof of principle for potential future applications, we proceeded with analyses of the animals’ learning and social behavior. Crucially, the ‘conservative detection’ dataset we subjected to the analysis here cannot satisfy the ambition of comprehensive long-term monitoring, but it is not merely a momentary snapshot of behavior either. Rather, we present a set of randomly selected behavioral samples that are fairly evenly distributed across space and time, and we will treat them as such.

### 3.2 Proof-of-principle application: investigation of social learning in mice

#### 3.2.1 Preference for high-reward probability options

After assessing the technical capabilities and limitations of the LMT-IC system, we proceeded to exemplify the method in a co-learning experiment in mice. In each IC operant corner, mice could obtain a liquid reward during the nightly trial phases either with a low (0.1) or high probability (0.9). Mice were randomly assigned to either team or solo learning configuration, and the reward probability distribution shifted every 3 d (individually for the ‘solo learning’ mice, congruently for the ‘team learning’ mice; Figure 3).

**Figure 9:**
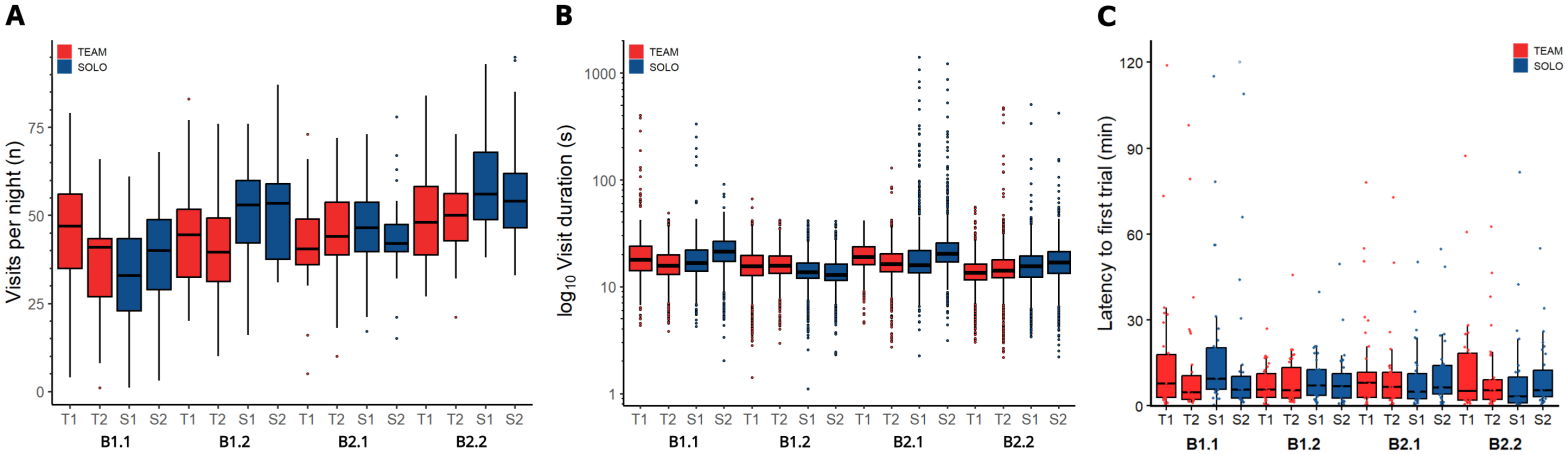
Number, duration and latency of operant corner visits per night. Lines represent median; lower hinges represent 0.25 quantiles; upper hinges represent 0.75 quantiles; whiskers represent ≤ Q3 + 1.5 × IQR and ≥ Q1–1.5 × IQR, respectively. Color-coding represents animal assignment to social learning configuration: red represents team-learning configuration, blue represents solo-learning configuration. **(A)** Number of visits to operant corners per night per animal, **(B)** visit duration depicted on log10 scale, and **(C)** latency in minutes until the first trial of a night.

**Figure 10:**
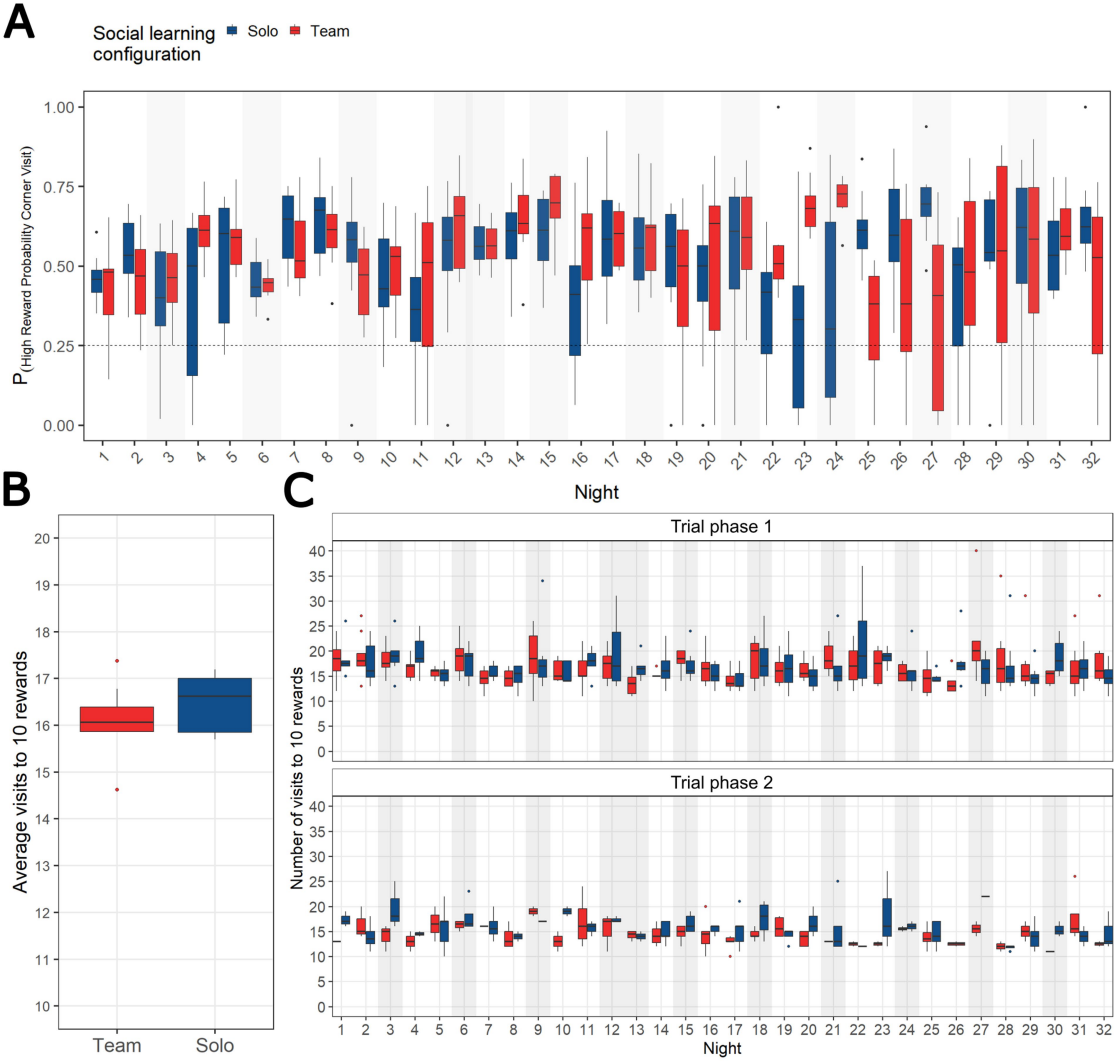
Preference for high reward probability corner (*p* **=** 0.9) in solo- and team-learning configuration. Animals lived continuously in the LMT-IC for the duration of the experiment and performed rewarded trials in 2 phases (herein, trial phases) each night. Every third night, the distribution of reward probabilities changed, and the animals had to adapt. Depicted is data from 16 animals tested in groups of *n* = 4. For team learners (*n* = 8), the distribution of reward probabilities was congruent at any given time, whereas for solo learners, the distribution of reward probabilities differed from all other animals in the group. **(A)** Average preference for the high-reward-probability option over time. The *y*-axis depicts the average preference per night for the high-reward probability corner, and the *x*-axis depicts the days of the experiment. The horizontal dashed line marks chance level (i.e., one of the 4 corners). Gray underlays indicate the nights on which a change of reward probability distribution occurred. **(B)** Average number of visits per trial phase until 10 rewards were received. **(C)** Number of visits until 10 rewards were received per phase 1 (21:00–23:00) and phase 2 (03:00–05:00) per night. The *y*-axis depicts the average number of visits until solo- (blue) and team-learning (red) animals collected 10 rewards per capita; the *x*-axis depicts the days of the experiment. Gray underlays indicate the nights on which a change of reward probability distribution occurred.

Per night, each animal visited the operant corners an average of 46.29 ± 15.59 times to participate in trials (Figure 9A), with the average visit lasting 18.56 ± 21.86 s (Figure 9B). Overall, animals readily entered the tubes leading to the operant corners, with a median latency to the first trial of the night of 6.12 min after operant corners became accessible (Figure 9C). We compared the development of a preference for the highest-reward-probability opportunities in either learning configuration by grouping the proportions of nightly visits to low- and high-reward-probability corners, respectively, across the experiment duration (Figure 10A) and modeling the proportions of visits to high-reward-probability corners by social learning configuration. Overall, our model estimated that co-learning (0.529 [0.450–0.608]) did not significantly increase preference for the highest reward probability option compared to individual learning (0.501 [0.422–0.579]; *p* = 0.5982). Animals from both social learning configurations, however, developed a preference (in 28.2 ± 3.51/32 nights) for the high-reward-probability options above chance level (0.25). Co-learning animals were also not estimated to adapt significantly more quickly to changing reward probabilities. An analysis of the average number of visits per animal to operant corners to accumulate 10 rewards in a phase estimated no significant difference (*p* = 0.1906) between team-learning (15.8 [15.4–16.3]) and solo-learning animals (16.2 [15.8–16.7]; Figures 10B,C).

#### 3.2.2 Testing for potential biases in interaction rates

**Figure 11:**
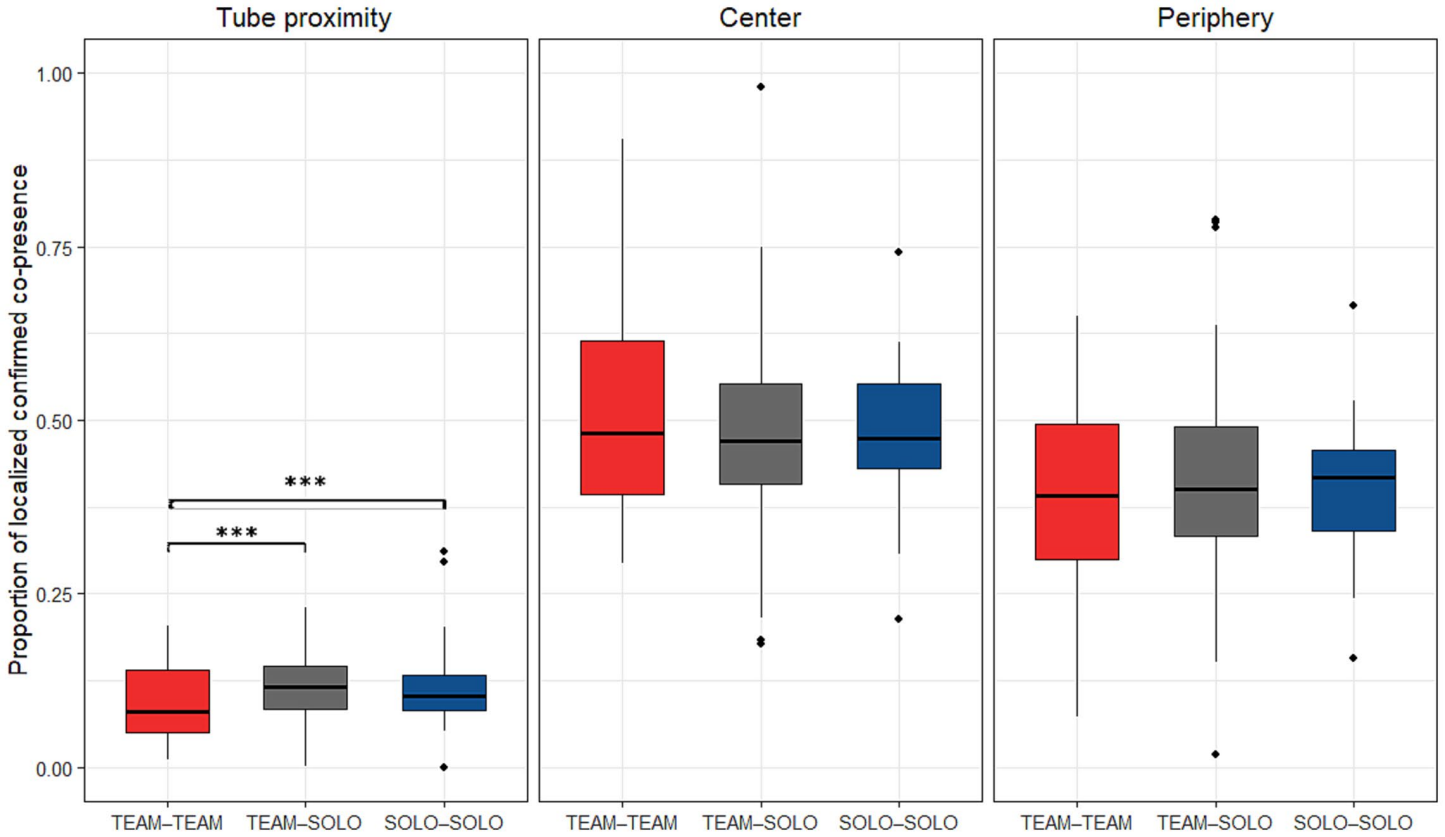
Relative localization of confirmed co-presence. For each frame with confirmed co-presence of dyads, the spatial localization in the cage was retrieved from the LMT SQLite databases and divided into three coarse spatial categories. A radius of 7.5 cm around tube entrances was defined as ‘tube proximity’, a central area of 34.56 × 34.56 cm was defined as ‘center’, and the remaining areas (in the peripheral margin, but outside the 7.5 cm radius around tube entrances) were defined as ‘periphery’. The fraction of frames with co-presence spent in the respective spatial categories was plotted, grouped by the social learning configuration of the co-present animals. The center line indicates the median, boxplots indicate the IQR, and whiskers represent 1.5 × IQR. Asterisks indicate confidence levels: *p ≤ 0.05, **p ≤ 0.01, ***p ≤ 0.001.

Due to the experimental design, co-learning animals that shared a congruent reward probability distribution may interact more often with one another than with other cagemates, by frequenting the same high-reward probability locations. To investigate whether co-learning animals interact more frequently near the entrances to tubes leading to the IC operant corners, we first examined the localization of confirmed co-presence intervals between all animals in a social group (Figure 11). During the co-learning experiment, the estimated probability (with 95% confidence intervals [CI] in square brackets) of at least one ‘team’-mouse being near a tube entrance during confirmed co-presence with its teammate was only 0.226 [0.173–0.290]. This is very similar to the estimated probability of at least one animal being localized to a tube entrance during the confirmed co-presence of team-solo dyads (0.223 [0.170–0.286]) and solo-solo dyads (0.223 [0.170–0.287]). We did model-estimate team-team dyads to be significantly more often co-present with at least one animal around tube entrances (*p* < 0.0001) than other pairings, but only by a slight margin in probability (0.003). Regardless of social learning configuration, the majority of confirmed co-presence between cagemates was spent in the cage center or periphery (Figure 11). When examining the probability of animals being co-present at the same tube entrance, the probabilities for all dyads were vanishingly small (T–T: 0.003 [0.002–0.006]; T-S: 0.003 [0.002–0.006]; S-S: 0.007 [0.004–0.012]) with minor, albeit significant differences between the tube-adjacent co-presence of solo-solo versus team-team (*p* < 0.0001) and team-solo (*p* < 0.0001) dyads, respectively. The minor increase in probability of ‘team’-animals being co-present at tube entrances compared to ‘solo’-animals led us to rule out potential differences in social interaction rates being solely explained by stochastic proximity.

#### 3.2.3 Dyadic interactions in team and solo-learning mice

**Figure 12:**
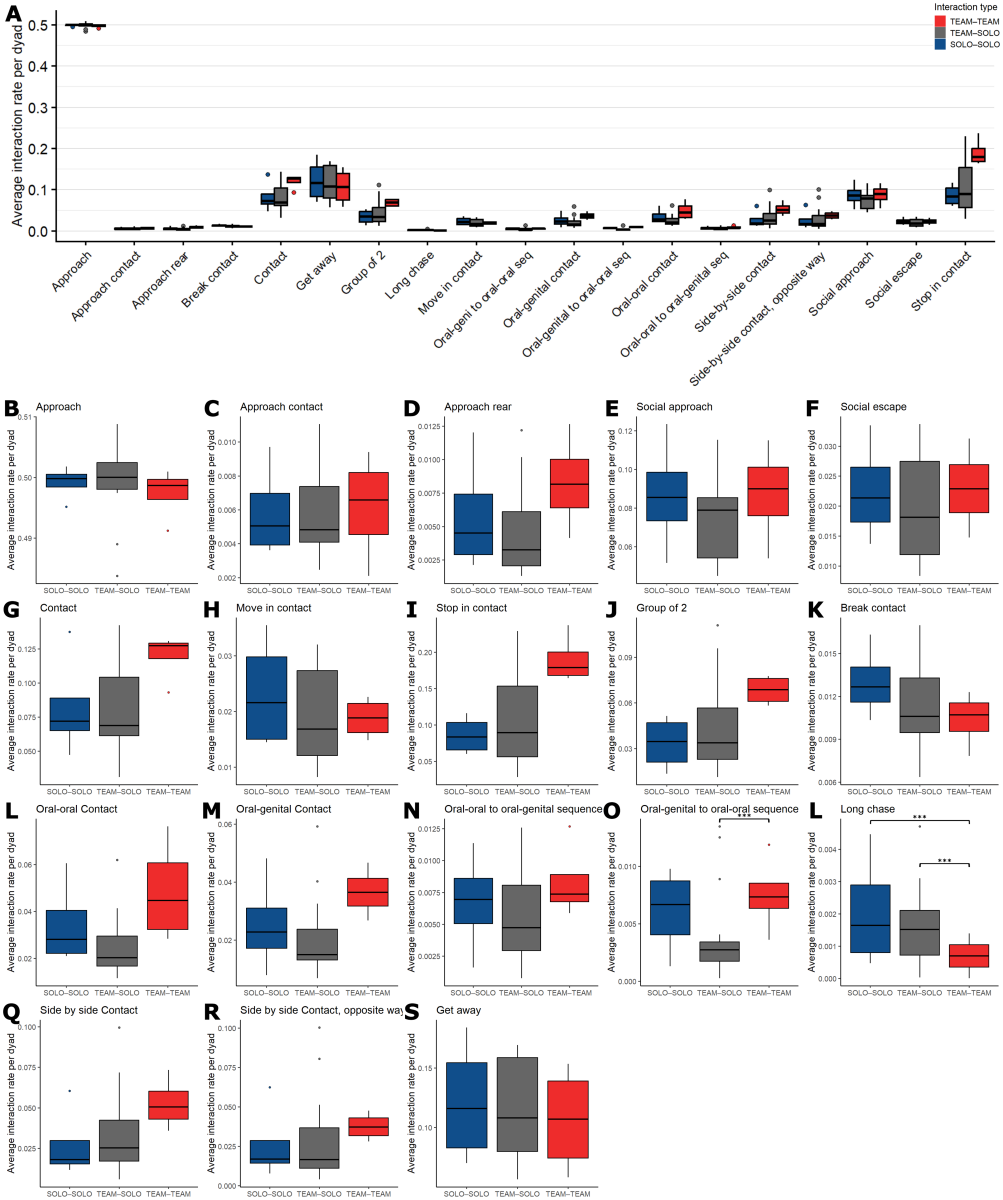
Average dyadic interaction rates per confirmed co-presence frame. Boxplots depict averaged dyadic interaction rates. To minimize ID inaccuracies, only tracks were considered that (1) contained at least 1 successful RFID read, (2) were within the maximum speed limit (100 cm/s), and (3) were above the movement threshold (1 pixel per consecutive frame, herein p/f, for >60 s). These criteria had to be met by all interaction partners for the event to be considered. Lines represent the median; lower hinges represent 0.25 quantiles; upper hinges represent 0.75 quantiles; whiskers represent ≤ Q3 + 1.5 × IQR and ≥ Q1–1.5 × IQR, respectively. Color-coding represents the categories of interaction between social learning configurations: solo-solo (blue), team-solo (gray), and team-team (red). *Y*-axes represent the average interaction rates per dyad: the total interaction durations in frames for all event types were calculated per dyad, as well as the confirmed co-presence in frames for all dyads. The ratio of total interaction duration / confirmed co-presence per dyad represents the interaction rate. Asterisks indicate confidence levels: *p ≤ 0.05, **p ≤ 0.01, ***p ≤ 0.001. **(A)** Overview plot. Along the *x*-axis, the observed dyadic interactions were plotted on a uniform linear *y*-axis. **(B–S)** Individual plots per interaction type with individual y-scales for comparison of the social learning configuration classes: **(B)** Approach, **(C)** approach contact, **(D)** approach rear, **(E)** social approach, **(F)** social escape, **(G)** contact, **(H)** move in contact, **(I)** stop in contact, **(J)** group of 2, **(K)** break contact, **(L)** oral-oral contact, **(M)** oral-genital contact, **(N)** oral-oral to oral-genital contact sequence, **(O)** oral-genital to oral-oral contact sequence, **(P)** long chase, **(Q)** side-by-side contact, **(R)** side-by-side contact in opposite orientation, **(S)** get away.

To investigate whether co-learning or individually learning animals differed in their social interactions, we fit a linear mixed-effects model to the log-transformed interaction rates per dyad. We observed a highly significant effect of the interaction type on the response variable (Figure 12A; *F* = 138.456; *p* < 2.2 × 10^−16^). Interaction rates are reported as back-transformed estimated marginal means, expressed as event frames per confirmed co-presence frame, with 95% confidence intervals in square brackets. Animals approaching each other was by far the most common interaction, estimated to occur nearly every other frame of confirmed co-presence (0.499 [0.367–0.677]). This was true for all animals regardless of learning configuration (T–T: 0.497 [0.270–0.917]; T-S: 0.499 [0.368–0.678]; S-S: 0.499 [0.271–0.920]). Animals were also estimated to partake in stationary contact (0.111 [0.082–0.151]) and distancing themselves from another (0.106 [0.078–0.144]) relatively frequently. However, most interaction types were estimated to occur at much lower rates per confirmed co-presence frame (Figure 12A). Notably, events defined by prolonged or more complex interactions, such as approaches of rearing animals (0.0051 [0.0038–0.0069]), long chase sequences (0.0006 [0.00039–0.0008]), or sequences of transitions between oral-oral to oral-genital contact (0.0058 [0.0043–0.0079]) and the inverse (0.0044 [0.0033–0.006]), were estimated to occur at lower rates. This could in part be due to the LMT detection interval filtering rationale, which favors shorter detection intervals (Figure 7) and, conversely, shorter dyadic interactions. However, these complex interactions were also observed to a lesser extent in the pre-processed annotated tracking data, suggesting an inherently lower rate.

Social learning configuration did not significantly affect the average dyadic interaction duration (*F* = 2.37, *p* = 0.12), but crucially impacted the rates of certain interaction types, as evidenced by a significant interaction of event type × learning configuration (Figures 12B–S; *F* = 1.523, *p* = 0.029). Co-learning animals were estimated to engage in oral-genital contact followed by oral-oral contact (oral-genital to oral-oral sequence) more frequently with teammates than with solo-learning animals (T–T: 0.007 [0.004–0.013]; T-S: 0.003 [0.002–0.003]; *p* = 0.013). Dyadic chase sequences (long chase) were more often estimated to involve at least one solo-learning animal (9.54 × 10^−4^ [6.96 × 10^−4^ - 0.001]) than only team-learners (1.24 × 10^−4^ [5.28 × 10^−5^ - 2.91 × 10^−4^]; T–T vs. T-S *p* = 3.94 × 10^−5^). Solo-exclusive chases were estimated to occur even more frequently (0.001 [7.93 × 10^−^4 - 0.003]; T–T vs. S-S *p* = 1.54 × 10^−5^). Oral-genital and oral-oral contact represent pro-social behaviors (Wang et al., 2026), whereas chasing behavior in female rodents is usually associated with aggressive or antagonistic behavior (Takahashi and Miczek, 2015). The significant effect of interaction type × social learning configuration was exclusive to the co-learning phase of the experiment. During the initial baseline measurement of learning performance, no significant effects of the future randomly assigned social learning configuration (*F* = 1.21; *p* = 0.321) or the interaction of prospective social learning configuration × interaction type on dyadic interaction rates were observed (*F* = 0.914; *p* = 0.614; Supplementary Figure 5). Importantly, these significant differences in interaction rates between team and solo-learning animals were estimated from only sporadic events, and for the majority of interaction types, no differences between social learning configurations were found. Furthermore, model estimates were based on a small subfraction of behavioral data. We therefore emphasize that we do not conclude that putatively socio-positive interactions occur among co-learning animals at an increased rate. Rather, these observations from a small fraction of the vast amount of data obtainable by the LMT-IC system serve to give a glimpse of the analytic potential of a full tracking dataset generated with a refined method.

### 3.3 Exploratory correlation of individual preference for high-reward options with social behavior

Following the analysis of dyadic interaction rates, we examined whether learning performance (i.e., preference for the high-reward-probability option) was linked to individual-level behaviors. To this end, we checked for monotonic association between nightly individual learning performance and behaviors that could be linked to prosocial or socially aversive interactions (Table 3) using Spearman’s rank correlation. A significant correlation between preference for the high-high-reward-probability options and social interactions was only observed sporadically, with the strongest correlation being established between the preference for the highest reward probability option for one individually learning animal (B1.1.S2) and ‘rearing in contact’ (*ϱ* = 0.52, *p* = 0.0033), which arguably represents a socio-positive interaction. Another strong correlation was observed in a team-learning animal within the same social group (B1.1.T1) between preference for the high-reward-probability corner and contact with other animals (ϱ = 0.5, *p* = 0.0045). For this animal, we observed the most associations between learning performance and socio-positive behaviors (approach: ϱ = 0.36, *p* = 0.0443; approach rear: ϱ = 0.44, *p* = 0.0129; contact: ϱ = 0.5, p = 0.0045; move in contact: ϱ = 0.44, *p* = 0.014; oral-oral contact: ϱ = 0.38, *p* = 0.0353; side-by-side contact: ϱ = 0.42, *p* = 0.0198; side-by-side contact in opposite orientation: ϱ = 0.45, *p* = 0.0113; social approach: ϱ = 0.41, *p* = 0.0213; and stop in contact: ϱ = 0.41, *p* = 0.0231), but also with aversive behaviors (rear isolated: ϱ = 0.39, *p* = 0.0296 and social escape: ϱ = 0.39, *p* = 0.0291). Overall, we found a monotonic association between nightly learning performance and 11/20 probed behaviors in animal B1.1.T1.

**Table 3:** Assessment of monotonic association between individual learning performance and different social behaviors using Spearman’s rank correlation.

Behavior	Metric	B1.1			B1.2				B2.1				B2.2				
		T1	T2	S1	S2	T1	T2	S1	S2	T1	T2	S1	S2	T1	T2	S1	S2
Approach	*n*	**31**	31	31	**30**	32	32	32	32	32	32	32	32	32	32	32	32
	*ϱ*	**0.36**	0.01	−0.17	**0.37**	−0.06	−0.16	−0.11	−0.05	−0.20	−0.28	0.11	0.32	0.12	0.17	−0.03	0.24
	*p*	**0.04**	0.98	0.36	**0.05**	0.76	0.38	0.56	0.81	0.27	0.13	0.55	0.08	0.53	0.37	0.88	0.19
Approach contact	*n*	31	31	31	30	32	32	32	32	32	32	32	**32**	32	32	32	32
	*ϱ*	0.32	0.06	−0.16	0.30	−0.14	−0.26	−0.23	0.08	−0.15	−0.15	0.12	**0.35**	−0.04	0.11	−0.05	−0.11
	*p*	0.08	0.75	0.39	0.10	0.46	0.14	0.21	0.68	0.42	0.40	0.52	**0.05**	0.83	0.54	0.79	0.57
Approach rear	*n*	**31**	31	31	**30**	32	32	32	32	32	32	32	32	32	32	32	32
	*ϱ*	**0.44**	0.06	−0.15	**0.40**	−0.09	−0.15	−0.27	0.17	−0.13	−0.02	0.14	0.08	−0.02	0.08	−0.13	−0.14
	*p*	**0.01**	0.75	0.42	**0.03**	0.64	0.43	0.14	0.37	0.47	0.90	0.45	0.68	0.92	0.68	0.49	0.44
Break contact	*n*	31	31	31	**30**	32	**32**	32	32	32	32	32	**32**	32	32	32	32
	*ϱ*	0.26	0.02	−0.10	**0.44**	−0.16	**−0.37**	−0.18	−0.15	−0.22	−0.15	0.16	**0.36**	0.04	0.14	−0.05	0.13
	*p*	0.16	0.91	0.61	**0.02**	0.37	**0.03**	0.32	0.40	0.23	0.42	0.37	**0.04**	0.83	0.46	0.78	0.49
Contact	*n*	**31**	31	31	30	32	32	32	32	32	32	32	32	32	32	32	32
	*ϱ*	**0.50**	−0.12	−0.14	0.30	0.07	−0.21	−0.21	−0.13	−0.16	−0.22	0.15	0.10	0.03	0.02	−0.01	0.06
	*p*	**0.005**	0.51	0.44	0.11	0.71	0.24	0.26	0.48	0.39	0.24	0.41	0.61	0.89	0.92	0.94	0.75
Get away	*n*	31	31	31	30	32	32	32	32	32	32	32	32	32	32	32	32
	*ϱ*	0.31	0.00	−0.13	0.35	−0.15	−0.24	−0.21	0.24	−0.18	−0.24	0.00	0.26	−0.06	0.14	−0.12	−0.20
	*p*	0.09	0.99	0.49	0.06	0.42	0.19	0.24	0.18	0.33	0.19	0.99	0.15	0.74	0.44	0.50	0.29
Group of 2	*n*	31	31	31	30	32	32	**32**	32	32	32	32	32	32	32	32	32
	*ϱ*	0.34	−0.19	−0.11	0.32	0.06	−0.33	**−0.35**	0.06	−0.22	−0.13	0.10	0.20	−0.04	0.20	−0.12	−0.21
	*p*	0.06	0.31	0.57	0.09	0.76	0.06	**0.05**	0.76	0.23	0.50	0.58	0.28	0.82	0.28	0.52	0.25
Move in contact	*n*	**31**	31	31	**30**	32	**32**	32	32	32	32	32	32	32	32	32	32
	*ϱ*	**0.44**	0.05	−0.02	**0.41**	−0.07	**−0.44**	−0.29	0.07	−0.21	−0.10	0.13	0.14	0.20	0.04	−0.04	0.06
	*p*	**0.01**	0.81	0.92	**0.02**	0.72	**0.01**	0.11	0.70	0.24	0.59	0.49	0.44	0.28	0.84	0.85	0.73
Move isolated	*n*	31	31	31	30	32	32	32	32	32	32	32	32	32	32	32	32
	*ϱ*	0.23	−0.12	−0.05	0.33	−0.12	−0.19	−0.21	0.30	−0.12	−0.22	−0.06	0.12	0.20	0.20	−0.05	0.19
	*p*	0.21	0.54	0.79	0.08	0.51	0.30	0.26	0.10	0.53	0.23	0.73	0.53	0.27	0.28	0.80	0.30
Nest of 3	*n*	31	31	31	30	32	32	32	32	32	32	32	32	32	**32**	32	32
	*ϱ*	0.10	−0.02	−0.26	0.25	0.03	−0.19	−0.07	0.21	−0.22	0.08	−0.05	0.01	0.15	**0.36**	−0.05	−0.27
	*p*	0.58	0.90	0.16	0.18	0.86	0.30	0.70	0.26	0.24	0.68	0.81	0.94	0.43	**0.04**	0.78	0.13
Oral-genital contact	*n*	31	31	31	30	32	32	32	32	32	32	32	32	32	32	32	32
	*ϱ*	0.33	−0.13	−0.16	0.26	−0.11	−0.34	−0.24	0.09	−0.24	0.10	0.04	0.15	0.06	−0.12	0.03	−0.02
	*p*	0.07	0.49	0.41	0.17	0.53	0.06	0.18	0.62	0.19	0.59	0.84	0.41	0.75	0.51	0.87	0.94
Oral-oral contact	*n*	**31**	31	31	30	32	32	32	32	32	32	32	32	32	32	32	32
	*ϱ*	**0.38**	0.15	−0.11	0.36	0.14	−0.21	−0.14	0.07	−0.13	−0.31	0.16	0.18	0.10	0.02	0.02	−0.02
	*p*	**0.04**	0.41	0.56	0.053	0.44	0.24	0.46	0.72	0.46	0.09	0.39	0.33	0.60	0.90	0.90	0.94
Rear in contact	*n*	31	31	31	**30**	32	**32**	32	32	32	32	32	32	32	32	32	32
	*ϱ*	0.32	0.07	−0.11	**0.52**	−0.21	**−0.42**	−0.16	0.18	−0.06	−0.29	0.22	0.21	−0.08	0.19	−0.07	−0.11
	*p*	0.08	0.71	0.56	**0.003**	0.24	**0.02**	0.40	0.33	0.75	0.11	0.24	0.25	0.66	0.30	0.71	0.55
Rear isolated	*n*	**31**	31	31	**30**	32	32	32	32	32	32	32	32	32	32	32	32
	*ϱ*	**0.39**	0.01	−0.14	**0.38**	−0.12	−0.07	−0.14	0.17	−0.23	−0.28	0.09	0.08	0.05	0.34	0.07	−0.32
	*p*	**0.03**	0.94	0.46	**0.04**	0.52	0.69	0.43	0.35	0.21	0.13	0.63	0.66	0.80	0.06	0.70	0.07
Side-by-side contact	*n*	**31**	31	31	30	32	32	32	32	32	32	32	32	32	32	32	32
	*ϱ*	**0.42**	−0.14	−0.14	0.33	0.22	−0.18	−0.18	−0.03	−0.11	−0.13	−0.14	0.06	−0.02	−0.19	−0.06	−0.03
	*p*	**0.02**	0.47	0.46	0.08	0.23	0.32	0.32	0.87	0.56	0.47	0.44	0.75	0.90	0.29	0.75	0.86
Side-by-side, opposite	*n*	**31**	31	31	30	32	32	32	32	32	32	32	32	32	32	32	32
	*ϱ*	**0.45**	0.06	−0.19	0.28	0.15	−0.16	−0.24	−0.07	−0.10	−0.13	0.32	0.19	−0.09	−0.08	0.00	0.03
	*p*	**0.01**	0.77	0.31	0.13	0.42	0.40	0.19	0.71	0.59	0.46	0.07	0.30	0.65	0.65	1.00	0.89
Social approach	*n*	**31**	31	31	30	32	32	32	32	32	32	32	32	32	32	32	32
	*ϱ*	**0.41**	−0.15	−0.12	0.34	−0.06	−0.27	−0.17	0.12	−0.26	−0.12	0.06	0.18	−0.20	0.06	−0.14	−0.19
	*p*	**0.02**	0.41	0.51	0.07	0.76	0.13	0.36	0.51	0.15	0.51	0.75	0.32	0.27	0.73	0.43	0.30
Social escape	*n*	**31**	31	31	**30**	32	**32**	32	32	32	32	32	32	32	32	32	32
	*ϱ*	**0.39**	−0.09	−0.04	**0.38**	−0.15	**−0.36**	−0.21	0.19	−0.18	−0.03	0.16	0.16	−0.07	0.06	−0.14	−0.17
	*p*	**0.03**	0.64	0.81	**0.04**	0.41	**0.04**	0.26	0.29	0.32	0.86	0.38	0.39	0.71	0.73	0.44	0.36
Stop in contact	*n*	**31**	31	31	30	32	32	32	32	32	32	32	32	32	32	32	32
	*ϱ*	**0.41**	−0.10	−0.17	0.28	−0.02	−0.19	−0.26	0.03	−0.19	−0.19	0.14	0.15	−0.17	0.09	−0.08	−0.22
	*p*	**0.02**	0.59	0.37	0.14	0.90	0.30	0.14	0.88	0.29	0.30	0.44	0.41	0.36	0.62	0.65	0.22
Stop isolated	*n*	31	31	31	**30**	32	32	32	32	32	32	32	32	32	32	32	32
	*ϱ*	0.19	0.00	−0.15	**0.40**	−0.12	−0.25	−0.08	0.14	−0.12	−0.17	0.13	−0.01	−0.04	0.20	−0.14	−0.21
	*p*	0.30	1.00	0.42	**0.03**	0.51	0.17	0.65	0.46	0.52	0.35	0.49	0.97	0.82	0.26	0.44	0.25

The total durations per night of automatically annotated social interactions were correlated with the nightly preference for the high-reward-probability option for all individual animals in four social groups (B1.1, B1.2, B2.1, B2.2). Animals assigned to the co-learning configuration are labeled T1 and T2; individual learning configuration animals are labeled S1 and S2. *n* represents the number of learning- and behavioral data pairings, which was derived from the number of nights during which a behavior could be confirmed according to Figure 4. *ϱ* represents Spearman’s Rho with the *p*-value *p* (95% bootstrap-CI). Statistically significant correlations are depicted in bold.

However, this observation did not hold true for the other team-learning animals. For the remaining co-learning mice, only a sporadic correlation was observed between nightly preference for the best reward option and social behaviors. For animal B1.2.T2, four behaviors were associated with nightly learning performance, all negatively (break contact: ϱ = − 0.37, *p* = 0.0348; move in contact: ϱ = −0.44, *p* = 0.0128; rear in contact: ϱ = −0.42, *p* = 0.0162; social escape: ϱ = −0.357, *p* = 0.0448), strikingly opposite to animal B1.1.T1. Beyond that, sporadic monotonic associations between learning and social interactions were observed for the individually learning animals, with only B1.1.S2 standing out with 8/20 probed behaviors correlated with nightly learning performance (approach: ϱ = 0.37, *p* = 0.0474; approach rear: ϱ = 0.4, *p* = 0.0284; break contact: ϱ = 0.44, *p* = 0.0154; move in contact: ϱ = 0.41, *p* = 0.0235; rear in contact: ϱ = 0.52, p = 0.0033; as well as rear in isolation: ϱ = 0.38, *p* = 0.0393). Crucially, the animal with the most associations between socio-positive behaviors and learning performance (team-learning animal B1.1.T1) expressed the second-lowest overall preference for the best reward option (Supplementary Figure 6; 0.385 ± 0.254). Only the individually learning animal B1.1.S2, for which a monotonic association was found for 8/20 behaviors, demonstrated lower learning performance (0.374 ± 0.196). The observation of numerous monotonic associations between nightly social interactions and preference for the best reward option only in the two ‘worst-performing’ animals may suggest that socio-positive behavior actually counteracts learning success. Of note, however, as we did not observe a negative correlation with such behaviors in the animals with the highest learning performance, this more likely reflects individuality in the animals rather than a general effect and should be interpreted with caution due to the restrictive criteria for behavioral data inclusion. Overall, no consistent association between learning performance and any individual behavior or social learning configuration could be established within the scope of our proof-of-principle examination.

In conclusion, we demonstrated a nearly fully automated method for presenting place-learning tasks to socially housed mice in a superenriched environment, while recording and annotating individual and social behaviors. We found behavioral annotation to be reliable, but individual identification was severely hindered by substantial visual occlusion from the video-tracking component. We mitigated this by introducing a data-inclusion rationale based on several layers of filters applied to tracking data. This approach proved effective in ensuring identity certainty and did not substantially bias the dataset in temporal or spatial distribution, but it only allowed us to consider a small subset of the gathered behavioral data. We exemplified this method in a probabilistic social learning paradigm, where reward probabilities continuously shifted and had to be relearned by the animals, either individually or in ‘teams’. We did not observe the co-learning setting to facilitate learning performance within the scope of the study, but found co- and individually learning animals to differ in some sporadic social interactions. Team learners engaged more often in sequences of oral-genital and oral-oral contact than their cagemates, and solo-learners were more often involved in chases. Monotonic associations between recorded behaviors and learning outcomes were only observed sporadically, and no overall link between social interactions and learning performance was established. While conclusions on the interplay between social interactions and spatial learning in mice are premature, we use this exemplary application of the LMT-IC method to illustrate potential future research avenues and highlight the need for further optimization, such as reducing opportunities for animal occlusion through enrichment. Once refined, this method may help to further elucidate social learning in mice, as well as other research questions.

## 4 Discussion

Here we present a method that integrates a widely used commercial system for automated behavioral testing (IntelliCage; TSE Systems, Berlin, Germany) and a cost-effective, easy-to-build open-source solution for 24/7 animal tracking and behavioral annotation (Live Mouse Tracker; de Chaumont et al., 2019) to achieve automated investigation of social learning and behaviors in mice. Combining the two apparatuses into a single system is achievable through straightforward modifications, with the main concern being to reduce RFID interference to a level allowing RFID antennae in both apparatuses to achieve reliable animal identification. We accomplished this by simply placing the IC operant corners outside the living area and connecting them to the cage via 40-cm PMMA tubes. Animals readily and willingly entered the tubes, with no apparent increase in aversion to participating in trials in the operant corners compared to the standard IC configuration, where operant corners are placed inside the cage area.

This paper reports on a method still being optimized. While we observed some differences in social interactions between animals assigned to co-learning and individual-learning configurations, these did not result in significant differences in learning success. We anticipate the method’s full potential will be realized only once obstacles to reliable animal identification are overcome. The system’s greatest current limitation lies in the identification accuracy of the live-tracking component in a heavily enriched area, as employed in this study, which allows animals to disappear from the camera’s field of view. This results in subpar detection rates (0.543 ± 0.232 overall during dark phases). Notably, we observed slightly higher overall detection rates for two social groups (B1.2 and B2.2) than for the other two (B1.1 and B2.1). This grouping correlates with the LMT-IC systems deployed in the study. While this may be coincidental, and it is conceivable that the social groups differed in hiding behavior and therefore occlusion from the camera, this observation suggests that one of the hardware systems performed more reliably in tracking than the other. At this time, we cannot offer a conclusive explanation for this observation, as both systems were constructed to the same standard. Regardless of the ultimate cause, this highlights the need for precise antenna tuning and prolonged testing of system accuracy and functionality, even during ongoing studies.

Animal occlusion most severely impacted the animal identification rate. Without all animals present in the LMT’s field of view simultaneously, animal ID proved highly unreliable, with tracking masks commonly flipping between animals. IDs could only be reliably restored when an antenna contact was made long enough for the RFID tag to be read. Notably, de Chaumont et al. (2019), who reported a much higher rate of correct animal ID assignment (97.31%), in their study deployed no enrichment beyond (1) wooden bedding, (2) 5 plastic toy bricks, (3) 6 cotton rolls, (4) a few brown paper strips, and (5) a monobloc mouse house consisting of one bent piece of PMMA (de Chaumont et al., 2019). The latter was open on two sides and, being made of red PMMA, which is entirely transmissive to IR light, did not obstruct animal tracking in any way. Furthermore, no material sufficient to build a full nest (e.g., paper towels) was provided, and in contrast to the present study, the animals had no opportunity to leave the arena at any time. Hence, all four animals in each group were visible to the camera at all times, with no opportunity for any occlusion.

This setup is suitable for housing groups of mice for short periods, as in de Chaumont et al. (2019) (max. 3 d). In contrast, the present study, conducted at the German Centre for the Protection of Laboratory Animals (Bf3R, n.d.) housed animals in the LMT-IC apparatus for a total of 75 d (including habituation, baseline measurement, and experimental phases). This demanded a much higher standard of housing, including enrichment to ensure animal welfare over extended periods. As a result, we applied a preexisting live-tracking algorithm to a much more dynamic and enriched environment than previously tested (Ohayon et al., 2013; de Chaumont et al., 2019). This superenriched environment, where each animal also disappeared from the trackable area on average ~50×/d, presented a far greater challenge for the LMT component than in the de Chaumont et al. study, highlighting the current limitations of the method.

Confronted with animal identification issues, we initially attempted to computationally reconstruct misidentified animal detections from RFID readings. As the LMT assigns identities based on probability from its video tracking component and only selectively activates RFID antennae to confirm and correct assigned identities (de Chaumont et al., 2019), the RFID dataset proved too sparse for effective reconstruction of animal trajectories. Instead of dedicating more time and resources to a potentially fruitless endeavor, we opted to rely on the ‘confirmed’ dataset, which imposed a high standard for tracking data to be included in the analyses (Figure 4). We mainly ruled out ‘impossible’ animal movements caused by probabilistic flips of tracking masks between animals through filters of animal speed, limited misidentification of enrichment components by excluding long periods of stationarity, and required uninterrupted tracks with direct RFID confirmation for data to be considered.

Imposing strict criteria for tracking data inclusion inevitably bears multiple caveats. The most obvious is the neglect of large portions of behavioral data. Our prioritization of ID certainty over comprehensive data inclusion further introduced the risk of selection bias. To mitigate this, we compared the spatial distribution of detection densities in both the raw and filtered datasets and found the distribution of retained animal detections to be comparable to that in the original data. Another potential bias was introduced by the congruent reward probability distributions among team-learning animals, which may have led to increased interaction rates merely by them targeting the same high-reward probability corners. Our analyses suggested that confirmed co-presence of animal dyads in the immediate vicinity of operant corner accesses occurred rarely. While team-team dyads were slightly more likely to be co-present with one animal located at a tube entrance, they were not more likely than other dyad types to be co-present with both animals at the same tube entrance. While these results may not suffice to entirely rule out any effects of the congruent reward probability distributions in team-learning animals, a putative spatial bias did not translate into increased interaction rates among team-learning animals overall.

Similar to reconstructive efforts, our filtering rationale serves as a potential fallback for tracking datasets with unreliable animal identification. We are confident that greater tracking accuracy in future studies could be achieved by considerably more straightforward methods, namely: (1) decreasing the degree of enrichment to prevent occlusion of the animals by enrichment components (de Chaumont et al., 2019) or (2) using specialized enrichment and nesting material, which appears opaque to murine vision (Peirson et al., 2018) but is penetrable by infrared light. Both approaches would ensure greater stability in visual contact between the tracking camera and the animals, which in turn yielded better tracking coverage in other studies using the LMT (de Chaumont et al., 2019; Maisterrena et al., 2024).

Reliable automated live multi-animal tracking remains a challenge for researchers, especially when no external markings are applied to the animals. Pose estimators like DeepLabCut (Mathis et al., 2018) have achieved considerable reliability and widespread use (Mathis and Mathis, 2020), but require manual training. Recently, a promising new approach was introduced with the ‘Tailtag’, a simple 3D-printable ArUco code that can be attached to mouse tails. It is reported to allow for reliable tracking in social groups of up to 20 animals over 7 d, outperforming the LMT in its multiplexing capabilities (Coulombe et al., 2025). However, the Tailtag cannot overcome visual occlusion from the camera view caused by enrichment or structural components of the experimental setup, such as trial corners. Unlike the LMT, it relies on external markings, albeit less invasive ones than, for instance, bleaching of the fur (Ohayon et al., 2013). Yu et al. (2025) followed a different approach to achieve comprehensive, fully automated behavioral testing in mice. Their home-cage-assisted mouse behavioral innovation and testing system (HABITS) allows mice to engage in self-initiated complex cognitive training and trials from their home cage via an impressive, machine learning algorithm-controlled platform (Yu et al., 2025). The system is designed for high-throughput behavioral screening and will likely become an invaluable tool for many neuroscientific research questions. Nevertheless, because it uses standard cage racks for individually housed mice, it cannot capture the social interactions of dynamic groups of mice living in semi-naturalistic environments.

In contrast, Murphy et al. (2016, 2020) developed an automated home-cage-based system in which mice are socially housed. Murphy et al. (2016) trained mice to enter a chamber connected to their home cage, where they would become head-fixed and undergo mesoscale cortical imaging. Murphy et al. (2020) expanded the apparatus with behavioral tests via licking sensors in which the mice could engage during cortical imaging. This method offers the benefit of directly combining behavioral testing with neural imaging of socially housed mice. However, the method is tailored to address a different set of neuroscientific and behavioral questions, as it primarily measures individual neural correlates and behavioral responses in individual animals, albeit socially housed. Systems such as the LMT-IC presented here, which are optimized to capture group interactions over prolonged periods, may find their niche among home-cage-based automated tracking approaches. We anticipate future developments in the field of home-cage-based multi-animal tracking and automated behavioral testing with excitement and hope to offer a modest perspective on the current capabilities and limitations of markerless methods in highly enriched environments over prolonged time periods.

In the present study, we did not find co-learning to facilitate learning outcomes compared to individual learning. Other studies using IntelliCages to test spatial learning found that co-learning paradigms could even rescue the performance of amyloid-β precursor protein-mutants (APP.V717I), which exhibited Alzheimer’s disease-related cognitive impairment (Kiryk et al., 2011). More recently, Smirnov et al. (2024) used the IntelliCage to study behavioral contagion in rats and reported that introducing a water-deprived demonstrator rat into a group induced water-seeking behavior among cagemates, whereas this response was attenuated when using Krushinsky-Molodkina observer rats with impaired social behavior.

However, the experimental designs in those studies differed markedly from the present investigation. The task presented to the mice in the APP study was simpler, and the experimental design less complicated than the method deployed in the present investigation—for instance, all animals housed together had the exact same learning goals in the Kiryk et al. (2011) study. The study on behavioral contagion by Smirnov et al. (2024), on the other hand, used a highly motivated, designated demonstrator animal to provide clear social cues, which was not present in this form in the present co-learning experiment.

In the study presented here, the experimental design (including the limited number of operant corners per cage, the frequent changes of reward probabilities, and the highly dynamic environment of social groups housing both individually and co-learning animals) may have favored heuristic learning strategies among the animals (i.e., frequenting corners until a reward was received over orienting themselves based on their teammates). Hence, we argue that future studies with a slightly refined probabilistic place learning paradigm may be better equipped to detect subtle effects of co-learning on learning outcomes by decreasing the feasibility of ‘trial & error’ strategies, for instance, by increasing the ‘cost’ of a trial over merely restricting the time window for maximizing rewards.

The results of the present study may seem at odds with Kiryk et al. (2011); however, newer reports corroborate our observations that mice do not perform better in co-learning scenarios than in individual learning. Vezyrakis et al. (2025) observed that mice solved puzzles more successfully (60% solved) when presented with them individually in an arena than when attempting to solve them in a semi-naturalistic group setting (21.4% solved). Similar effects have been observed in other mammalian species (Benson-Amram et al., 2013), and are hypothesized to be caused by social environments distracting animals from tasks, as well as animals having less time and energy for problem-solving due to involvement in complex social interactions, including competition for space and potential mates (Vezyrakis et al., 2025; Kummer and Goodall, 1985; König and Lindholm, 2012). We also observed a monotonic association between learning performance and several socio-positive behaviors only in animals with the lowest overall preference for the best reward option; however, there was no overall negative association between socio-positive interactions and learning performance. Yet, the highly dynamic, ‘noisy’ social environment deployed in the present study may have prevented co-learning animals from utilizing available social cues to their advantage.

How social behaviors relate to learning in social interactions in mice remains elusive. Mice can learn by observation (Mainardi et al., 1988; Carlier and Jamon, 2006) and are influenced in their food choices by olfactory cues from conspecifics (reviewed by Lang et al., 2023). In the present study, mice could observe cagemates entering and leaving the tube entrances to operant corners, but trial outcomes—i.e., whether a mouse obtained a reward—were not immediately visible. Instead, they would need to rely primarily on olfactory cues to detect sucrose intake by a cagemate returning from a successful trial. Mice can olfactorily detect sucrose in liquid solutions (Zukerman et al., 2009; Glendinning et al., 2024), and social transmission of food preference (STFP) is a well-established paradigm in rodent social learning (Galef and Wigmore, 1983; Valsecchi and Galef, 1989). A recent study also demonstrated that mice showed a strong preference for the scent of conspecifics that had been rewarded with sucrose solution (Winiarski et al., 2025). While the Winiarski et al. (2025) study, in contrast to the present report, used bedding material from sucrose-rewarded mice, these findings still support the plausibility of mice developing place preferences based on sucrose-associated olfactory cues on conspecifics. We observed team animals engaging in oral-genital to oral-oral contact transitions more frequently than their solo-learning cagemates. While this behavior may have led to animals smelling sucrose residue on recently rewarded cagemates and thereby contributed to social learning, this was a sporadic observation among many interactions that did not differ between team- and solo-learning animals and, furthermore, did not manifest in overall increased learning success among the team-learning animals. In the current study, the ambiguous availability of visual and olfactory cues may have hindered the development of an advantage by exploiting social cues over individual learning.

In concert with the limitations of the behavioral analysis presented here, we see the need for further research to elucidate social learning in mice, which is likely a complex, multifactorial process that may not be fully understood through ‘classical’ temporally and spatially confined tests of dyadic interactions alone (Lang et al., 2023). We anticipate modeling of social interactions in conjunction with home-cage-based approaches combining RFID and video tracking to shed more light on social learning in mice in the near future (Shemesh et al., 2013). Once the current limitations of the present method are addressed, the LMT-IC may contribute to this development. We will continue to optimize the method and further our investigation of social learning with refined tools. We also aim to better understand social learning strategies by deploying reinforcement learning model approaches to mouse behavior in social groups. Predictions from such models can be easily tested in the multi-armed bandit-inspired probabilistic experimental design, demanding a trade-off between exploration and exploitation of the best known option.

Beyond basic research on social learning in the mouse model, we anticipate a wide array of possible applications that require both cognitive testing and behavioral tracking with minimal experimenter interference. For instance, it may be deployed for investigating genes associated with the onset of autism in animal models (Mineur et al., 2006; Leblond et al., 2012; de Chaumont et al., 2019) in more realistic group settings, or to screen novel compounds for adverse effects on cognition and social capabilities, which may only become apparent in prolonged group interactions. Overall, we will continue our efforts to further optimize this approach in order to contribute to the fast-growing field of automated home-cage-based behavioral investigation.

## Data Availability

The datasets presented in this study can be found in online repositories. The names of the repository/repositories and accession number(s) can be found at: EMBL-EBI BioStudies S-BSST2600, https://www.ebi.ac.uk/biostudies/studies/S-BSST2600.
