# Supplementary material for: Development of an automated approach for investigating social learning in mice
Source: Front Behav Neurosci. 2026 Jul 15;20:1789820. doi: 10.3389/fnbeh.2026.1789820 (PMC13416557; doi:10.3389/fnbeh.2026.1789820)
Supplement: Supplementary file 1 [file Data_Sheet_1.docx]

**Supplementary Information**

**
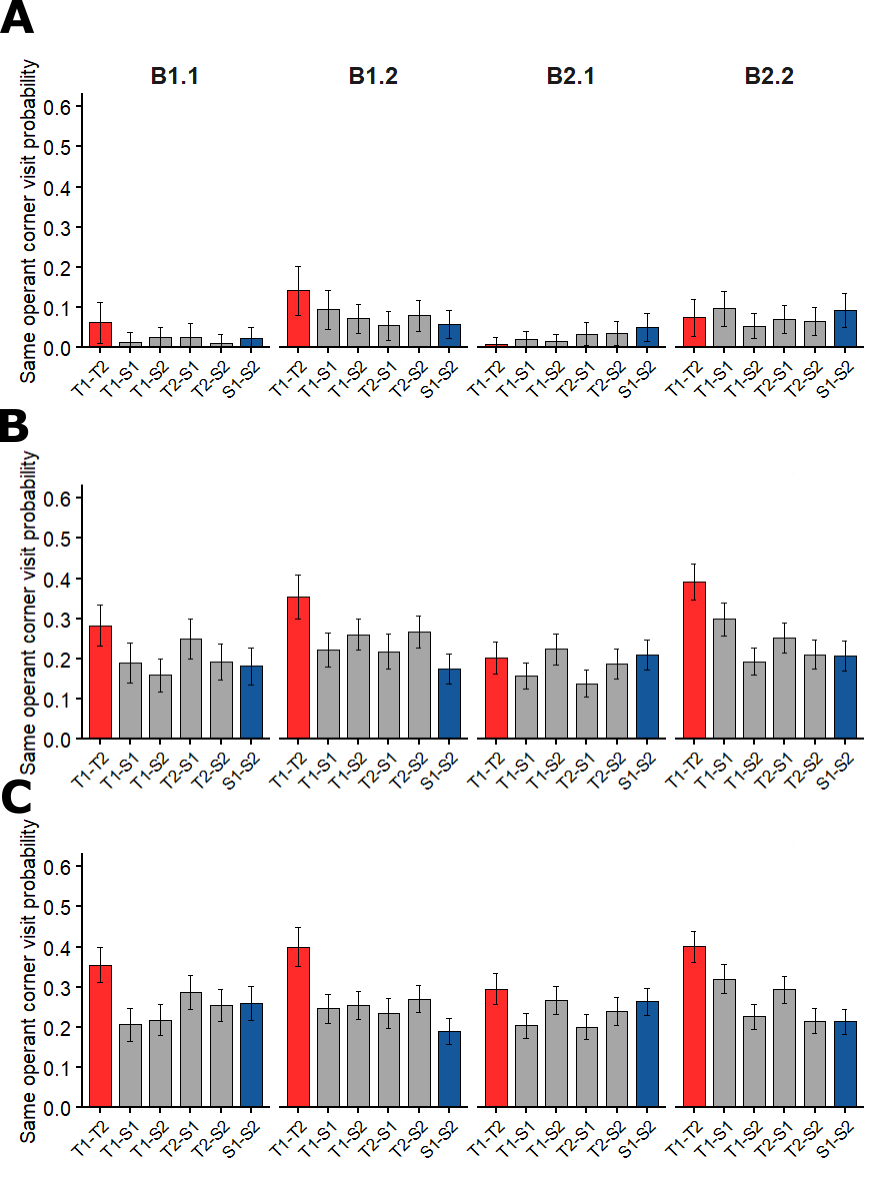
**

**Supplementary Figure 1:** Exploratory examination of same-corner sequential trials. For each trial, we calculated the probability of a trial occurring by a different animal per social group within (**A**) 10 s, (**B**) 30 s, and (**C**) 1 min. Sequential same-corner trials were summarized per animal dyad. Color coding indicates dyad configuration: team-team (red), team-solo (gray), and solo-solo (blue). Team dyads shared a highest-reward-probability corner, whereas all other dyads did not. While sequential same-corner trial probabilities varied between dyads (χ² = 70.31, p = 1.09 × 10⁻⁶), Holm-corrected pairwise comparisons indicated no significant contrasts between dyad pairs.

**
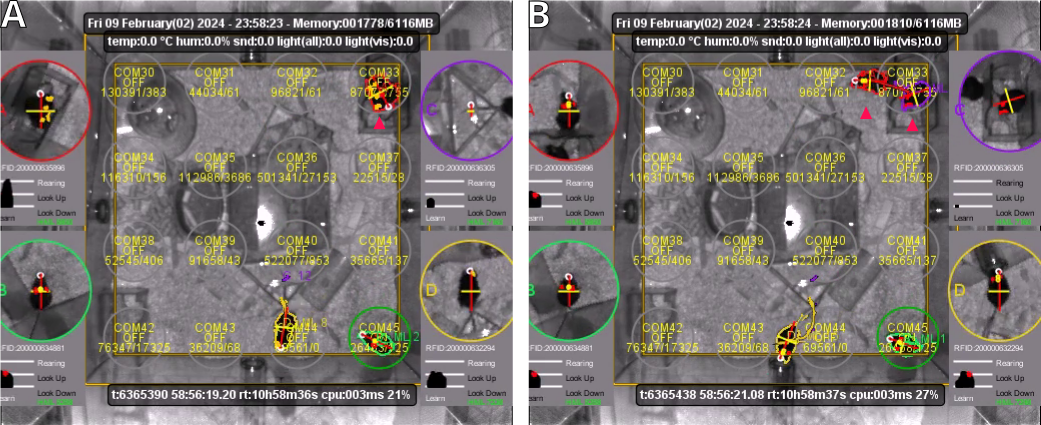
**

**Supplementary Figure 2: Exemplary identification failure of LMT.** Figure depicts two snapshots from the live tracking view, taken 1.6 s apart from each other. In both snapshots, all four animals of the social group (B2.2) are present in the field of view. In (**A**), animals S2 and T2 are sitting huddled together in the small house in the upper right corner, highlighted by the bright red triangle. The LMT fails to separate the two animals and erroneously applies the same (red) tracking mask to both of them. (**B**) Shortly after, animal S2 leaves the small house, creating some distance between itself and animal T2. The LMT is able to correctly identify both animals again, highlighted by two bright red triangles, and overlays two (red and purple) tracking masks.

**
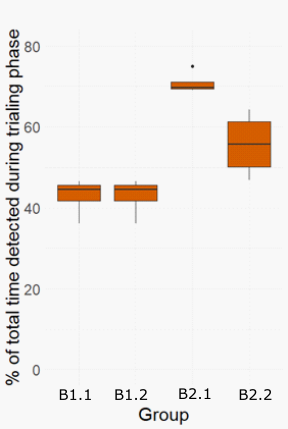
**

**Supplementary Figure 3:** **Exemplary detection rates during first trialing session of a night (21:00 – 23:00)**


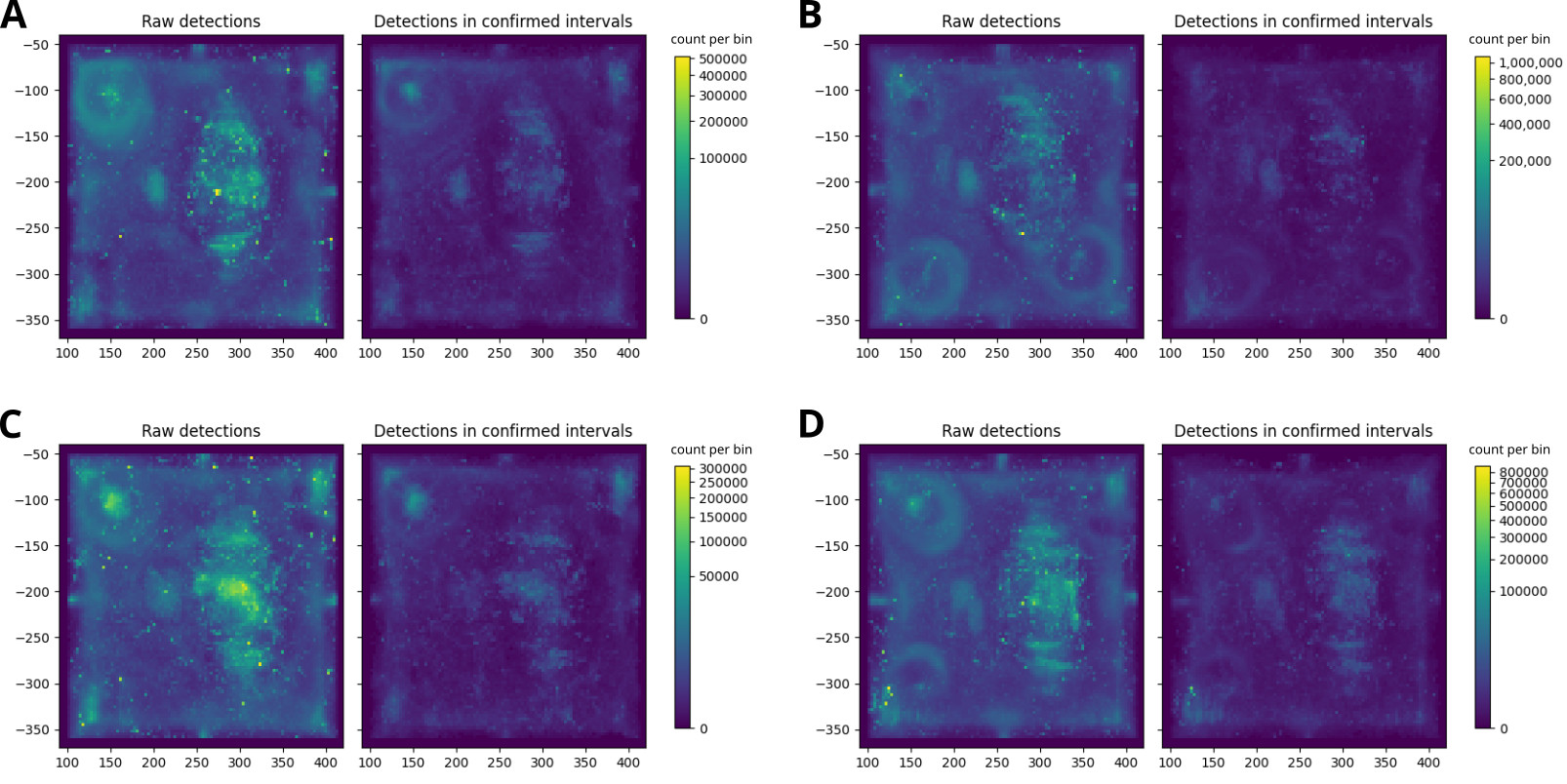


**Supplementary Figure 4: Detection heatmaps in raw and filtered LMT datasets.** All animal detections across baseline measurements and co-learning experiment were pooled per social group in the raw state and subsequent to the ‘detections in confirmed intervals’-filtering pipeline according to **Figure (4)**. The LMT field of view (corresponding to the entire living area) was divided into equal 100 × 100 bins and the absolute detection counts per bin were plotted with both raw and filtered datasets on the same color scale per social group. (**A**) B1.1 (**B**) B1.2 (**C**) B2.1 (**D**) B2.2.

**(Next page) Supplementary Figure. 5: Average dyadic interaction rates per confirmed copresence frame during baseline measurements.** Animals were not assigned to co-learning or individual learning configuration yet, all animals received individual tasks. Color-coding is retroactively projected from the later assignment to allow for direct comparison of the animal groups. Boxplots depict average dyadic interaction type. To minimize ID inaccuracies, only tracks were considered that 1) contained at least 1 successful RFID read and 2) were within the maximum speed limit (100 cm/s) as well as 3) above the movement threshold (1 px/f). These criteria had to be met by all interaction partners in order for the event to be considered. Lines represent median; lower hinges represent 0.25 quantiles; upper hinges represent 0.75 quantiles; whiskers represent ≤ Q3 + 1.5 × IQR and ≥ Q1 − 1.5 × IQR, respectively. Y-axes represent the average interaction rates per dyad: the total interaction durations in frames for all event types were calculated per dyad; as well as the confirmed copresence in frames for all dyads. The ratio of total interaction duration / confirmed copresence per dyad represents the interaction rate. (**A**) Overview plot. Along the x-axis, the observed dyadic interactions were plotted on a uniform linear y-axis. (**B**-**S**) Individual plots per interaction type with individual y-scales for comparison of the social learning configuration classes: (**B**) Approach (**C**) Approach contact (**D**) Approach rear (**E**) Social approach (**F**) Social escape (**G**) Contact (**H**) Move in contact (**I**) Stop in contact (**J**) Group of 2 (**K**) Break contact (**L**) Oral-oral contact (**M**) Oral-genital contact (**N**) Oral-oral to oral-genital contact sequence (**O**) Oral-genital to oral-oral contact sequence (**P**) Long chase (**Q**) Side by side contact (**R**) Side by side contact in opposite orientation (**S**) Get away.


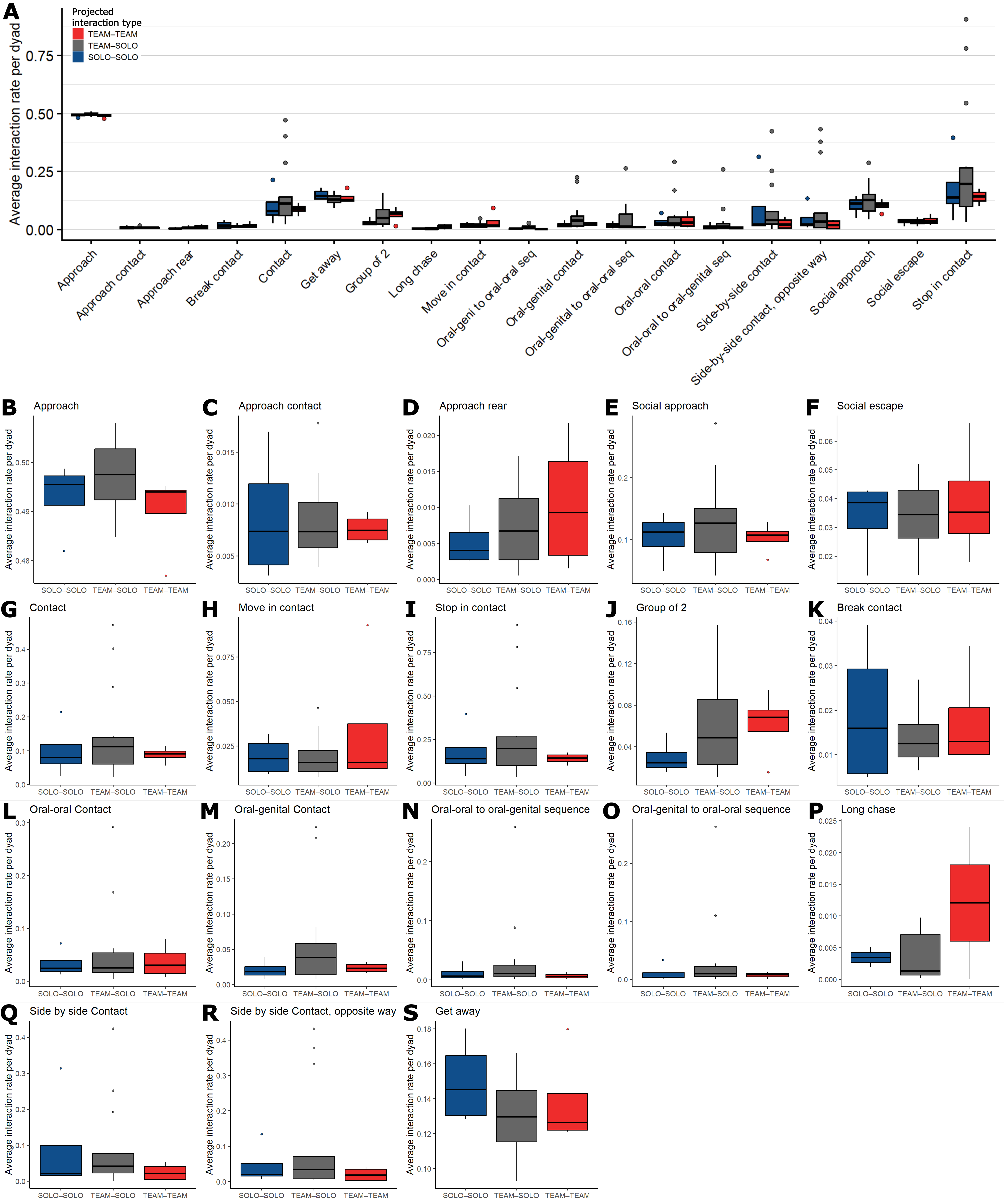


**
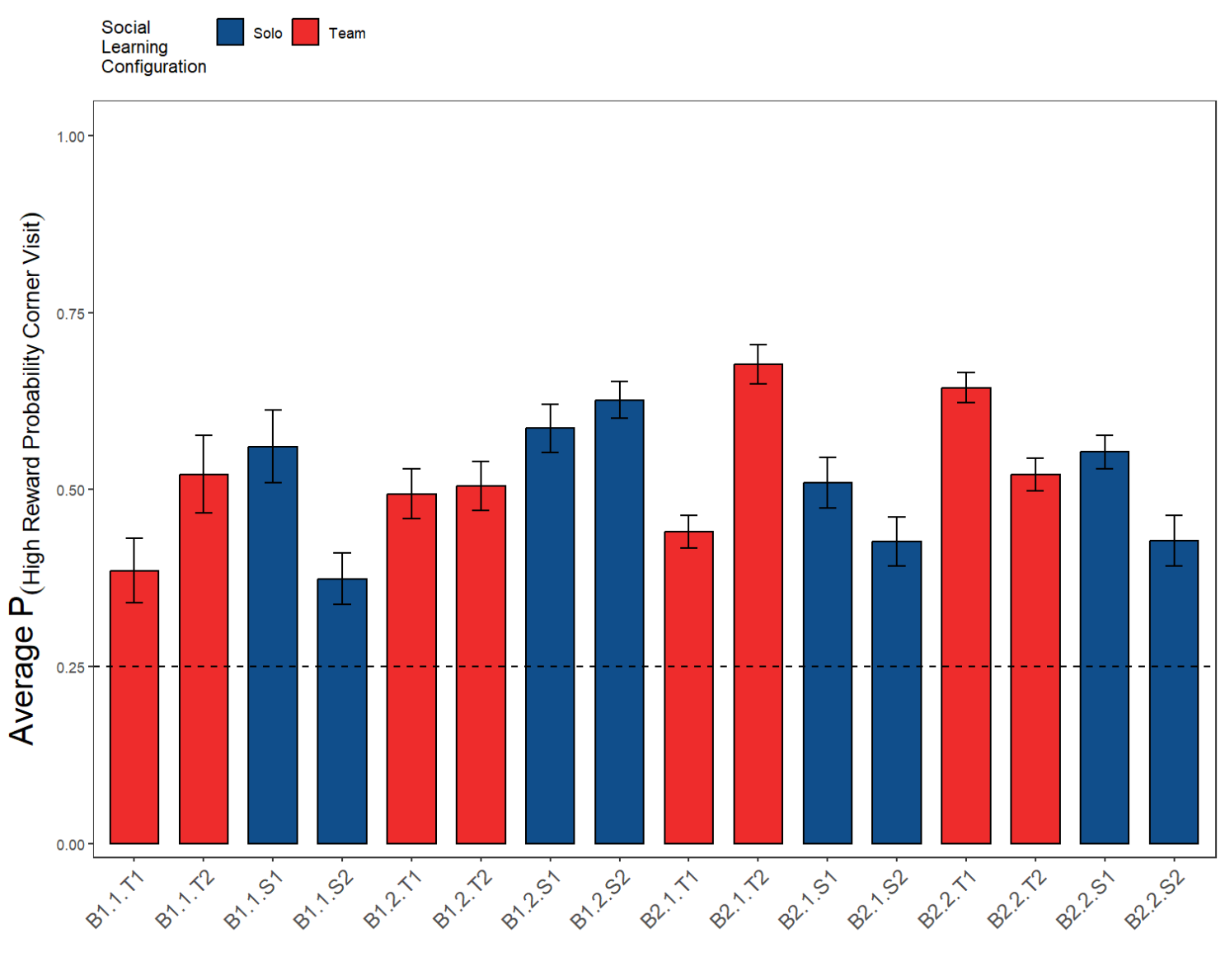
Supplementary Figure 6:** **Overall average preference for high reward probability corner per animal, sorted by batch (B1, B2) and social group (1, 2).** Batch 1 – group 1 (B1.1), batch 1 – group 2 (B1.2), batch 2 – group 1 (B2.1) and batch 2 - group 2 (B2.2) denominate the batches / social groups. T1 and T2 denominate the team-learning animals, S1 and S2 the solo-learning animals. The horizontal dashed line marks chance level (i.e., one of the 4 corners).
